# Supplementary material for: Dynamic stimulation promotes functional tissue-like organization of a 3D human lymphoid microenvironment model in vitro
Source: Cell Rep Methods. 2025 Jul 11;5(7):101105. doi: 10.1016/j.crmeth.2025.101105 (PMC12296514; doi:10.1016/j.crmeth.2025.101105)
Supplement: Document S1. Figures S1–S3 and Table S1 [file mmc1.pdf]

Cell Reports Methods, Volume *5*

Supplemental information

Dynamic stimulation promotes functional tissue-like organization of a 3D human lymphoid microenvironment model *in vitro*

Dafne Barozzi, Fiorella Scagnoli, Federica Barbaglio, Daniela Belloni, Davide Ribezzi, Silvia Farè, Valeria Berno, Riccardo Pinos, Marta Sampietro, Margherita Pauri, Barbara Vergani, Francesco Mantegazza, Paolo Ghia, and Cristina Scielzo

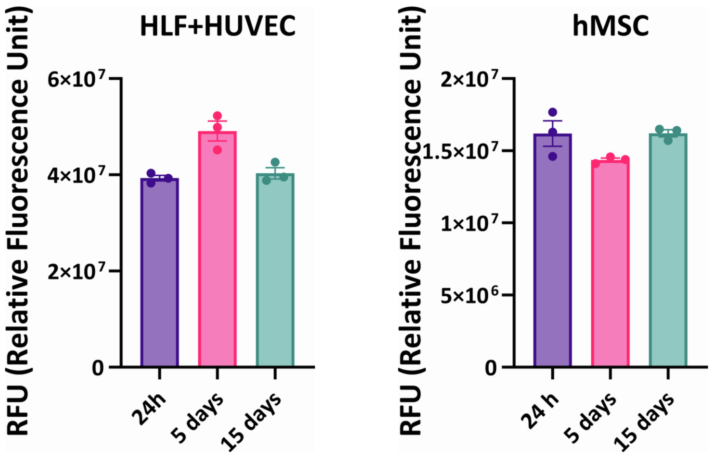

Supplementary Figure 1 – AlamarBlue viability assay, **related to Figure 1** AlamarBlue cell viability assay fluorescence intensity values showing metabolic activity of the 3D cultures over time (n=3). All data are represented by mean ± SEM.

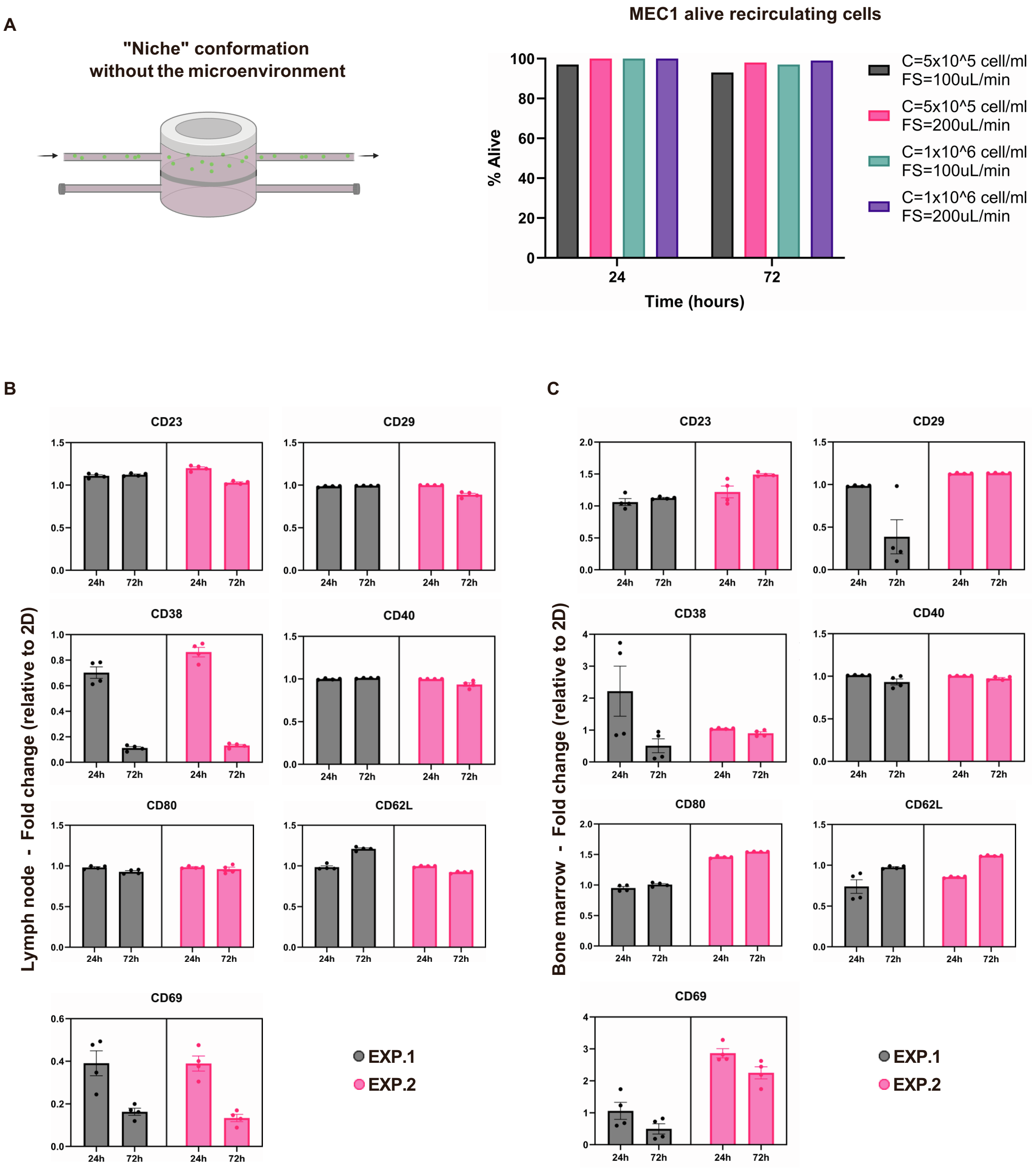

Supplementary Figure 2 - Recirculation of MEC1 cells in the system, related to Figure 7 (A) Representation of MEC1 cells recirculating in the "niche" conformation of the dynamic system without the microenvironment. The histogram shows circulating MEC1 cells viability at different cell concentration and flow rates without the microenvironment. (B) Fold change (relative to 2D condition) of different markers analyzed on MEC1 cells circulating in the dynamic sydtem with both microenvironments – bone marrow and lymph node. Data of two independent experiments (EXP.1 and EXP.2) are shown separately. For each experiment were performed 4 biological replicates. All data are represented by mean ± SEM.

A

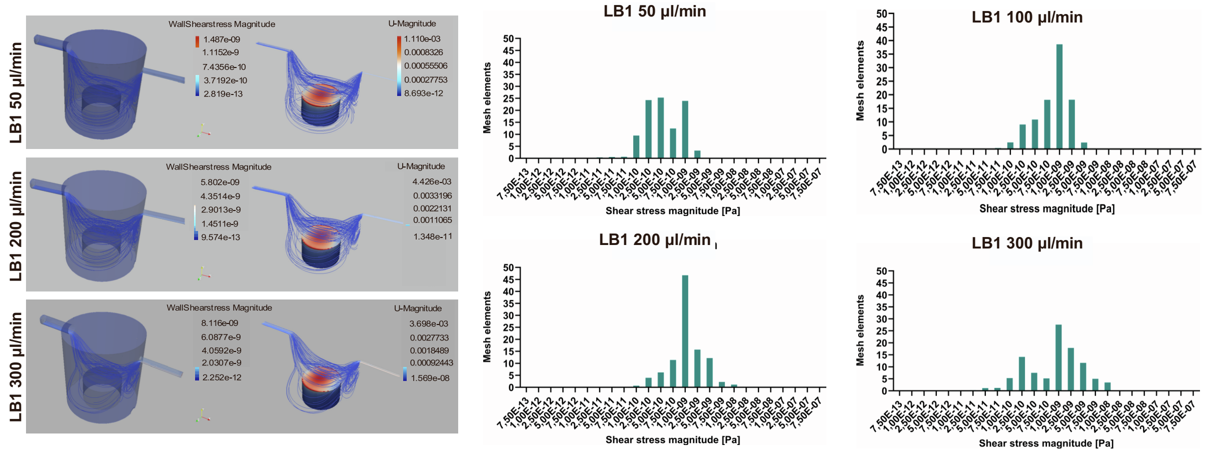

B

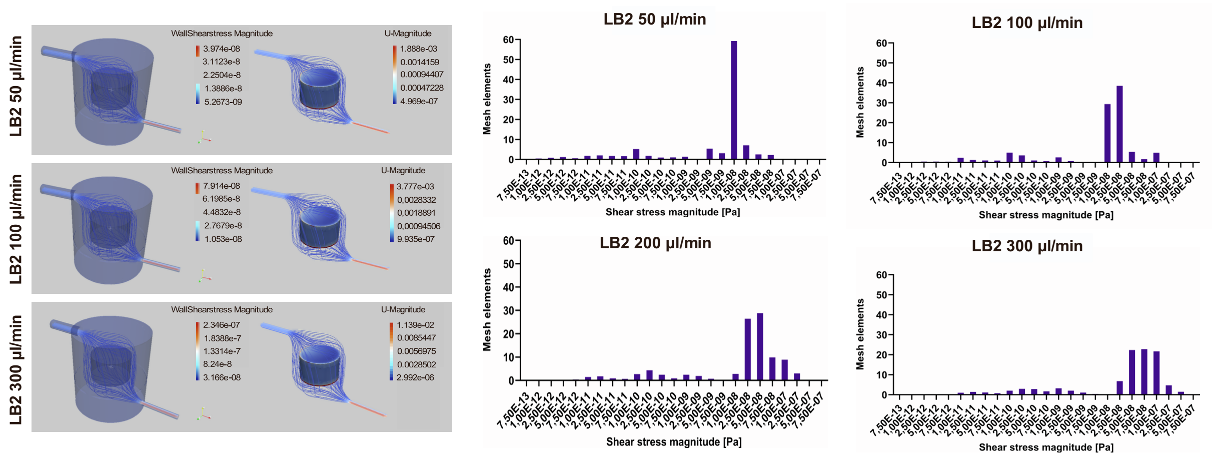

Supplementary Figure 3 - Computational Fluid Dynamic study, related to STAR methods "Computational Fluid Dynamic study (CFD)" section (A) Representation of flow lines in the bone marrow configuration (LB1) used for the bioreactors at flow rates different from the chosen ones. The colorimetric maps in both images represent the shear stress (left) and laminar flow rate (right). The histograms on the left represent the percentage of mesh elements (y-axis), normalized on the total number of the mesh elements related to the scaffold, characterized by a certain shear stress magnitude (x-axis, expressed in Pa) for LB1 at all flow rates. (B) Representation of flow lines in the lymph node configuration (LB2) used for the bioreactors at flow rates different from the chosen ones. The colorimetric maps in both images represent the shear stress (left) and laminar flow rate (right). The histograms on the left represent the percentage of mesh elements (y-axis), normalized on the total number of the mesh elements related to the scaffold, characterized by a certain shear stress magnitude (x-axis, expressed in Pa) for LB2 at all flow rates.

A

Bone Marrow	EXP.1 2D	EXP.1 24h	EXP.1 72h	EXP.2 2D	EXP.2 24h	EXP.2 72h
IgM	5713,45	3263,9975	3636,86	4376,88	17,7925	4247,5525
IgD	26016,07	24810,4625	17636,055	16525,24	0,6325	17989,6525
CD29	59238,16	13496,51	5689,5075	9099,6	98,315	16599,11
CD49d	24411,25	29323,65	26087,8	31389,34	77,72	28690,885
CD38	29835,25	31673,245	29760,0675	84809,27	8,7575	42017,625
CD44	47320,38	43186,37	27674,8175	42486,62	93,9975	29261,96
CD40	20958,2	37306,0325	16205,405	30221,43	99,385	18627,1175
CD80	25771,33	22743,025	27188,69	15749,12	89,89	34443,8575
CD45	26203,7	26192,0025	22351,16	41687,79	82,165	24694,0025
CXCR4	3734,54	5010,1125	4066,03	6193,17	20,44	4850,3825
CCR7	17842,02	18253,8575	17222,2375	21214,8	11,0025	19008,07
CD69	20851,49	20634,11	18321,7425	23592,9	25,88	18607,285
CD23	129048,02	150448,71	148565,915	148312,02	78,33	102356,04
CD62L	16293,49	9833,2975	16468,555	23894,45	61,0125	43379,3025

B

Lymph Node	EXP.1 2D	EXP.1 24h	EXP.1 72h	EXP.2 2D	EXP.2 24h	EXP.2 72h
IgM	9142,16	3327,41	4004,4625	6509,1	5672,92	3881,18
IgD	15739,23	35919,195	21038,7125	15155,32	16895,1975	18698,06
CD29	60886,2	12250,76	15231,005	39052,75	33210,355	8280,245
CD49d	38001,93	35668,1475	44880,14	38021,01	31303,005	29014,57
CD38	32474,07	29502,6225	29206,49	28028,86	25947,7725	26075,9375
CD44	30579,11	31131,35	19917,6675	213491,17	244533,315	23567,3425
CD40	19515,66	21571,8425	13464,3575	104447,41	143572,81	14600,1025
CD80	29601,01	21788,465	17087,46	29043,97	21524,4025	16095,4425
CD45	26242,6	20266,7225	21738,425	34207,05	38899,91	24543,02
CXCR4	3125,24	4679,6125	6400,85	35325,43	74773,7725	19825,1525
CCR7	17539,15	17366,7275	17874,43	22169	23738,12	20430,365
CD69	20714,79	18991,8625	18011,1325	19919,72	19073,245	19228,8725
CD23	167758,41	144428,0675	193795,29	125147,46	129598,66	112524,7825
CD62L	13620,56	11193,01	31932,67	89535,38	60702,49	34426,58

Supplementary Table 1 - Mean fluorescence intensities of flow cytometry analysis, related to Figure 7 Table reporting all the mean fluorescence intensities of the MEC1 cells recirculating in the bone marrow (A) and lymph node (B) systems. The reported values refer to the gated populations positive for the different markers displayed in Fig. 6D. Gates have been designed on FMO controls. MFI values in each experiment (EXP.1 and EXP.2) refers to the mean of the biological replicates MFI (n=4).
