# Supplementary material for: Dynamic stimulation promotes functional tissue-like organization of a 3D human lymphoid microenvironment model in vitro
Source: Cell Rep Methods. 2025 Jul 11;5(7):101105. doi: 10.1016/j.crmeth.2025.101105 (PMC12296514; doi:10.1016/j.crmeth.2025.101105)
Supplement: Document S2. Article plus supplemental information [file mmc4.pdf]

Cell Reports
Methods

Article

Dynamic stimulation promotes functional tissue-like organization of a 3D human lymphoid microenvironment model *in vitro*

Graphical abstract

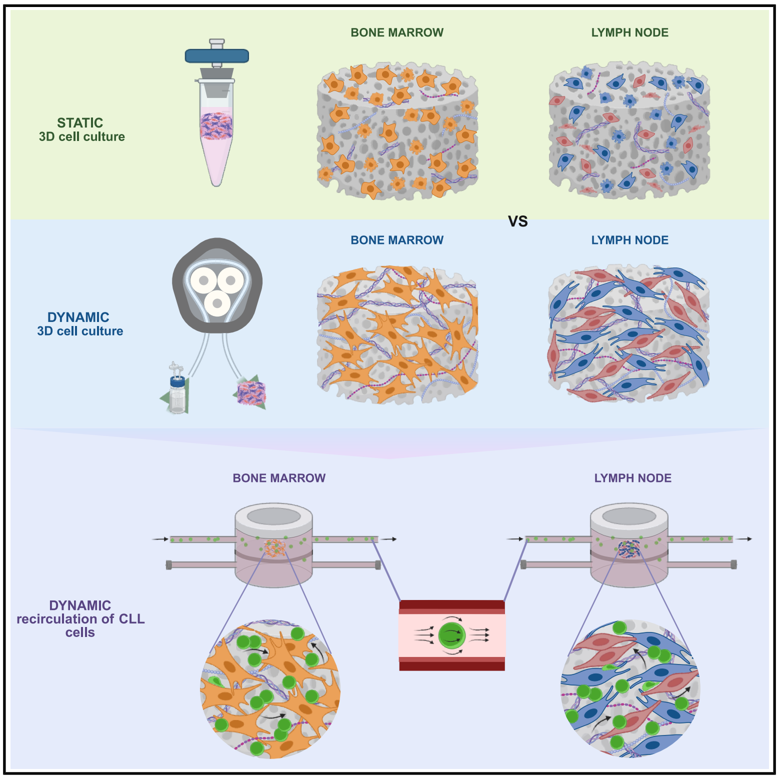

Authors

Dafne Barozzi, Fiorella Scagnoli, Federica Barbaglio, ..., Francesco Mantegazza, Paolo Ghia, Cristina Scielzo

Correspondence

scielzo.cristina@hsr.it

In brief

Three-dimensional (3D) *in vitro* modeling is opening the doors to high-quality *ex vivo* systems for the study of blood cancers and their interaction with the tissue microenvironment. Barozzi et al. develop a dynamic lymphoid-like model to study CLL physiopathology and show the advantages of the perfusion on 3D cell cultures.

Highlights

- Optimizing a 3D dynamic *in vitro* lymphoid-like model to study CLL pathogenesis
- Highlighting the advantages of perfusion stimulation on 3D cell cultures
- Assessing tissue-dependent changes of leukemic cells in dynamic settings

Barozzi et al., 2025, Cell Reports Methods *5*, 101105
July 21, 2025 © 2025 The Author(s). Published by Elsevier Inc.
https://doi.org/10.1016/j.crmeth.2025.101105

CellPress

Cell Reports Methods

CellPress
OPEN ACCESS

Article

Dynamic stimulation promotes functional tissue-like organization of a 3D human lymphoid microenvironment model *in vitro*

Dafne Barozzi,[1,2] Fiorella Scagnoli,[1] Federica Barbaglio,[1] Daniela Belloni,[1] Davide Ribezzi,[3,4] Silvia Farè,[3] Valeria Berno,[5] Riccardo Pinos,[1] Marta Sampietro,[1] Margherita Pauri,[1] Barbara Vergani,[2] Francesco Mantegazza,[2] Paolo Ghia,[6,7] and Cristina Scielzo[1,8,*]

[1]Division of Experimental Oncology, Malignant B Cells Biology and 3D Modelling Unit, IRCCS Ospedale San Raffaele, 20132 Milano, Italy
[2]School of Medicine and Surgery, Università degli Studi di Milano-Bicocca, Vedano al Lambro, 20854 Monza, Italy
[3]Department of Chemistry, Materials and Chemical Engineering, Politecnico di Milano, 20133 Milano, Italy
[4]Department of Orthopaedics, University Medical Center Utrecht, Utrecht University, 3584 CX Utrecht, the Netherlands
[5]ALEMBIC, Advanced Microscopy Laboratory, IRCCS Ospedale San Raffaele and Università Vita-Salute San Raffaele, 20132 Milano, Italy
[6]B-Cell Neoplasia Unit and Strategic Research Program on CLL, Division of Experimental Oncology, IRCCS Ospedale San Raffaele, 20132 Milano, Italy
[7]School of Medicine, Università Vita-Salute San Raffaele, 20132 Milano, Italy
[8]Lead contact
*Correspondence: scielzo.cristina@hsr.it
https://doi.org/10.1016/j.crmeth.2025.101105

MOTIVATION Three-dimensional (3D) bioengineered lymphoid tissues represent a rapidly expanding area of research due to their translational potential for studying both physiological and pathological conditions. In hematological cancer research, increasing attention is being paid to unraveling the complex interactions between cancer cells and the tumor microenvironment (TME). Most 3D models of blood cancers described in the literature, however, rely on scaffold-free methods under static conditions, often failing to incorporate the TME or capture the dynamic nature of hematological malignancies. In this study, we established and optimized tissue-like models of the human lymph node and bone marrow microenvironments using a dynamic perfusion system designed to recirculate leukemia cells.

SUMMARY

This work focused on generating a three-dimensional (3D) *in vitro* dynamic model to study chronic lymphocytic leukemia (CLL) cell dissemination, homing, and mechanisms of therapy resistance. We used a gelatin-based, hard porous biomaterial as a support matrix to develop 3D tissue-like models of the human lymph node and bone marrow, which were matured inside bioreactors under dynamic perfusion of medium. Comparing static and dynamic cultures of these 3D constructs revealed that perfusion promoted a tissue-like internal organization of cells, characterized by the expression of specific functional markers and deposition of an intricate extracellular matrix protein network. Recirculation of CLL cells within the dynamic system led to changes in leukemic cell behavior and in the expression of key markers involved in tumor progression. These findings suggest that the model is well suited for investigating the pathophysiological mechanisms of CLL and potentially other hematological malignancies.

INTRODUCTION

Our current understanding of physiological and pathophysiological processes is largely based on two-dimensional (2D) cell cultures and animal models. While 2D cultures remain the gold standard for *in vitro* studies due to their low cost, reproducibility, and suitability for high-throughput analyses, these "flat" models lack the multidimensional complexity of the *in vivo* environment.[1] Animal models provide the alternative of more complex systems but often fail to faithfully represent human pathophysiology and raise ethical and economic concerns.[2–4] By contrast, three-dimensional (3D) *in vitro* tissue modeling refers to the reconstruction of realistic microanatomy and tissue function under defined conditions, offering valuable insights into cell-cell and cell-matrix interactions. Indeed, the growing interest in *in vitro* tissue bioengineering in recent decades stems from its

© 2025 The Author(s). Published by Elsevier Inc.
This is an open access article under the CC BY license (http://creativecommons.org/licenses/by/4.0/).

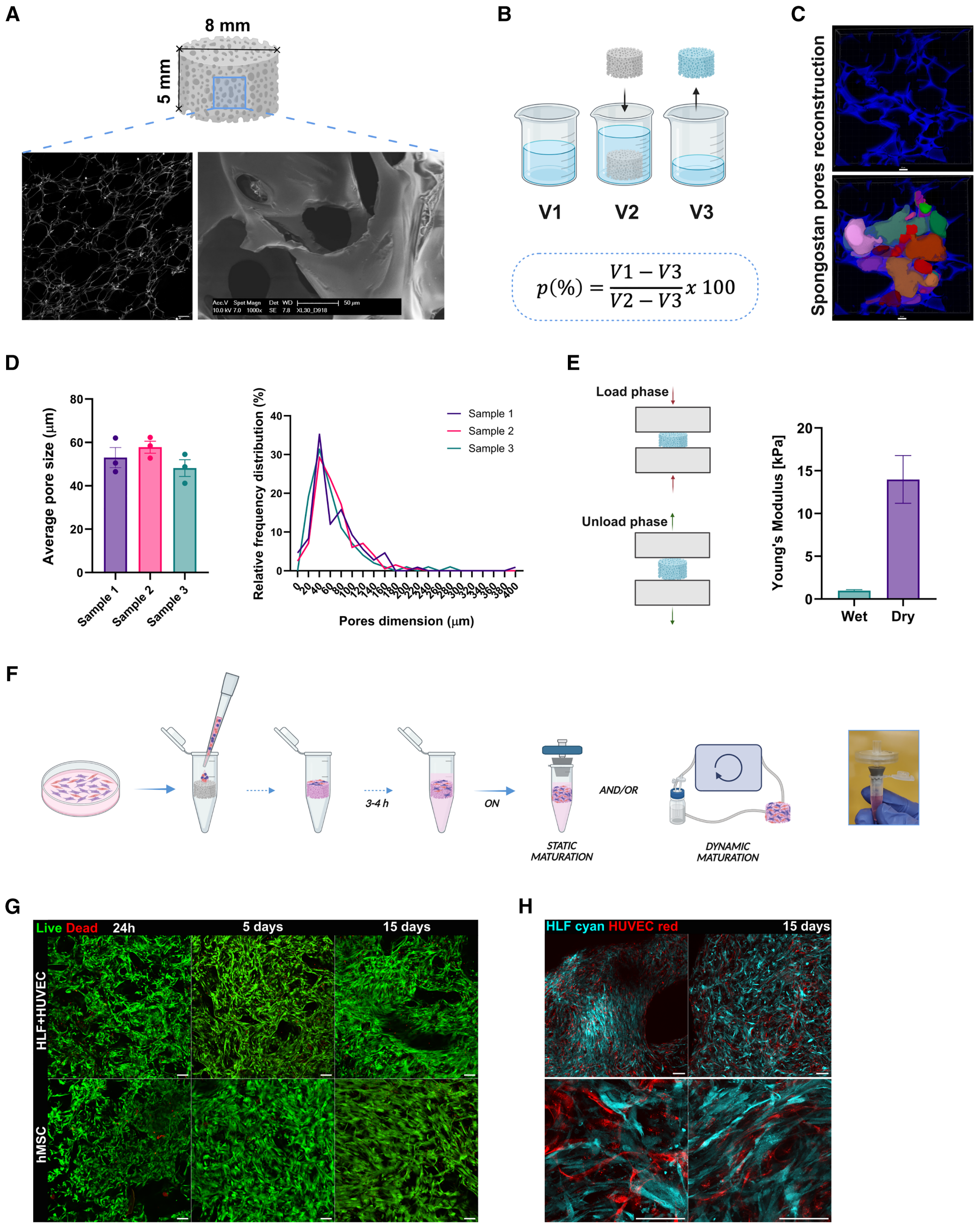

Figure 1. Biomaterial characterization and cell viability

(A) Spongostan scaffold dimensions and shape; biomaterial internal topography shown by second harmonic generation (SHG) imaging (scale bar, 200 μm) and scanning electron microscopy (SEM) (scale bar, 50 μm) images.

(B) Representation of liquid displacement method used to calculate the porosity of the biomaterial (n = 3).

(C) (Top) Maximum projection of z stack reconstructed images of a Spongostan slice. (Bottom) Example of the pore size analysis on Spongostan z stack confocal reconstructions through Arivis software. z Stacks were acquired using a 20× objective lens. Scale bars, 50 μm.

(D) (Left) Histogram showing the pore size weighted average of the same three independent scaffolds. (Right) Pore size distribution frequency analysis on the three independent samples. All data are represented by mean ± SEM.

(E) Mechanical characterization of Spongostan scaffold. (Left) Graphical representation of dynamic mechanical analyzer (DMA) compression measurement. (Right) Compressive modulus analysis of dry and wet Spongostan (n = 3).

(legend continued on next page)

ability to replicate the intricate and information-rich microenvironment of human tissues.[5] These models enable complex communication between different cell types and between cells and the extracellular matrix (ECM), offering more predictive data than traditional 2D cultures.[6]

Numerous studies have shown that cells cultured in 3D behave differently from those in 2D, as they more closely resemble *in vivo* conditions.[7] Crucially, the regulation of many cellular processes is influenced by the mechanical properties of the environment, including ECM stiffness and the shear stress generated by interstitial flow, blood, and lymphatic circulation vessels.[8–10] In cancer research, reproducing the functional and architectural features of specific tissues is essential for understanding disease mechanisms and predicting therapeutic responses. In modeling hematological malignancies, for example, incorporating diverse tissue microenvironments is critical, as blood cancers are heterogeneous and can originate in multiple anatomical sites, often with poorly defined origins.[11–13]

Chronic lymphocytic leukemia (CLL) is characterized by the accumulation of mature B cells in the peripheral blood and lymphoid organs. Despite advances in treatment, CLL remains incurable, largely due to its high inter- and intra-patient clonal heterogeneity.[14] CLL cells actively interact with lymphoid tissues, forming supportive and protective niches on which the activation and progression of the tumor strongly depends.[15–18] *In vitro* studies have shown that co-culture with stromal cells and specific soluble factors enhances the survival and proliferation of CLL cells, highlighting the importance of reproducing the tissue microenvironment.[19] We previously developed a long-term bioprinted 3D culture model of CLL, demonstrating that 3D architecture supports CLL cell viability.[20] We also established a 3D bone marrow (BM) model using a microgravity bioreactor that recapitulates CLL-BM stromal cell interactions and allows investigation of patient-specific responses to Bruton's tyrosine kinase (BTK) inhibition with ibrutinib, as well as the dynamic processes of CLL cell mobilization and homing.[21,22]

Several studies have reported the benefits of 3D dynamic modeling using mesenchymal stem cells (MSCs).[23] Few, however, have addressed more complex BM or lymph node (LN) tissue models, likely due to the inherent structural and cellular complexity of these tissues.[24,25] Indeed, most 3D models of hematological malignancies rely on scaffold-free techniques,[11] which are particularly challenging for non-adherent cell types such as blood cancer cells due to the difficulties in handling self-assembled structures. Moreover, many of these models fail to include the tissue microenvironment.

Despite the challenges, there is growing interest in engineering 3D lymphoid-like tissues, not only to study hematologic malignancies but also to investigate physiological processes such as hematopoiesis and immune responses.[26,27] Nonetheless, only a limited number of studies have reported on such models, and most have focused on specific components of the lymphoid niche,[28] organ-on-a-chip approaches,[29] or cell-coated multilayers in microfluidic channels.[30] Only a few of these incorporate dynamic systems to enhance tissue mimicry.[25,30,31] To address this gap, we aimed to generate dynamic 3D *in vitro* models of lymphoid-like microenvironments—specifically, LN and BM—to conduct functional studies on CLL cells. We focused on optimizing protocols for generating dynamic lymphoid tissue models and comparing their performance to conventional 2D and 3D static cultures. To create our models, we selected a biocompatible and biodegradable porcine gelatin-based hard porous scaffold,[32] namely Spongostan. This choice was based on our previous work, in which Spongostan supported the generation of CLL and multiple myeloma BM microenvironments.[21,22,33] A major advantage of this scaffold is its ability to facilitate efficient cell seeding and support the subsequent organization of cells into complex structures. Our objective was to more closely mimic the architecture and function of *in vivo* lymphoid tissues by recreating the journey of blood cells into and within these environments. To achieve this, we scaled up the tissue dimensions, employed an ECM-like support matrix to incorporate microenvironmental components. Numerous biophysical forces affect living organisms, including pressure and shear stress, which arises from frictional forces generated by blood or interstitial flow acting on vessel walls and within tissues.[10] Given the essential role of these forces in tissue morphogenesis and pathophysiology *in vivo*, we enhanced our static culture conditions by introducing a milli-fluidic dynamic component to generate interstitial-like flow within the 3D tissue constructs.[34,35]

RESULTS

Spongostan provides an ideal support for 3D LN and BM scaffolds

In our first analyses, we thoroughly characterized the biomaterial and observed that its internal topography consists of a highly heterogeneous network of open, interconnected pores that provide mechanical support for cell adhesion and growth (Figure 1A). These features are essential for promoting oxygen and nutrient exchange, cellular migration, proliferation, and differentiation.[32,36,37]

Using the liquid displacement method, we determined that Spongostan has a porosity of 76% (Figure 1B).[36] The 3D reconstructions of Spongostan slices using confocal microscopy revealed a heterogeneous distribution of micropores, with a weighted average diameter of 53 μm (Figures 1C and 1D). We additionally measured the compressive modulus of the acellular scaffold, which was 11 kPa when dry and 1 kPa when soaked in medium (Figure 1E). These values fall within the reported stiffness range for soft lymphatic tissues, including BM

(F) Schematic representation of the cells seeding process into Spongostan scaffold. (Left) Real image of the static setting: the filter is maintained as stable and the culture as sterile thanks to an adapter that was 3D printed in our laboratory.

(G) Live/dead fluorescent viability assay on Spongostan seeded with lymphatic fibroblasts with endothelial cells (HLF+HUVEC) and human mesenchymal stem cells (hMSCs), matured in static conditions (green = live cells, red = dead cells). z Stacks were acquired using a 10× objective lens. Scale bars, 100 μm.

(H) Confocal z stack reconstruction of HLF (cyan) and HUVEC (red) cells organizing inside the Spongostan scaffold in static conditions. In the first row, z stacks were acquired using a 10× objective lens. In the second row, z stacks were acquired using a 40× objective lens. Scale bars, 100 μm.

(0.25–24.7 kPa)[38] and secondary lymphoid organs (SLO; 2.30–7.81 kPa),[39,40] supporting the suitability of Spongostan for modeling these tissues.

The next step in protocol development involved selecting the relevant cell types. The BM and LN are architecturally and functionally complex tissues, composed of various compartmentalized cell populations.[11] Reproducing this multifaceted environment *in vitro* requires stepwise protocol optimization, introducing one variable at a time. To this end, we selected specific cell types as the starting point. For the BM model, we used human BM-derived MSCs (hMSCs), while the LN model incorporated human lymphatic fibroblasts (HLFs) and human umbilical vein endothelial cells (HUVECs). We assessed cell viability and proliferation for each cell type within the 3D scaffold, optimizing the seeding protocol (see STAR Methods; Figure 1F). In both models—BM (hMSC) and LN (HLF+HUVEC)—the cells exhibited a 3D elongated morphology and remained viable through day 15, as determined by live/dead fluorescence assay (Figure 1G). To evaluate cell-cell interactions, we used fluorescent nanoparticle-labeled HLFs and HUVECs and observed how these cells organized and interacted within the 3D scaffold (Figure 1H). In parallel, we performed an Alamar Blue assay at 24 h, 5 days, and 15 days post-seeding (Figure S1), which confirmed stable metabolic activity in both microenvironments over time. Together, these data support the suitability of Spongostan to develop tissue-like models as BM and LN, maintaining cell viability and allowing cell spreading and interactions in 3D.

Computational fluid dynamics analysis supports the design of the perfusion system for 3D scaffold culture

We next studied the effect of enhancing our 3D static model adding a flow perfusion system. To this end, we placed the scaffolds in two types of bioreactors (IVTech): LiveBox1 (LB1, Figure 2AI) and LiveBox2 (LB2, Figure 2AII), both connected to a peristaltic pump (LiveFlow, Figure 2AIII), which simulates the dynamic stimuli typical of *in vivo* environments. The pump ensured constant medium perfusion through the bioreactors and across the cell cultures (Figure 2A).

We selected different flow configurations for the BM and LN models. The BM model was cultured in the LB1 bioreactor at a flow rate of 100 μL/min, targeting primarily the external surfaces of the scaffold. Here, hMSCs in the BM scaffold exhibited good growth and organization. When we exposed HLF and HUVEC cells to the same conditions, we saw no elongation or organization. By contrast, exposing the entire LN model scaffold directly to flow in the LB2 bioreactor and perfusing at 200 μL/min under a sigmoidal flow pattern, we stimulated endothelial maturation and promoted the development of an LN-like architecture.

To better understand the physical forces acting within the perfusion system, we retrospectively modeled the experimental setup using computational fluid dynamics (CFD). We simulated a representative case involving a 5-mm-high, 8-mm-diameter Spongostan scaffold placed in LB1 (Figure 2BI) or in the upper chamber of LB2 (Figure 2BII), with respective flow rates of 100 and 200 μL/min. We first aimed to simulate the FD within the bioreactor circuits and analyze the distribution of shear stress across the scaffold to characterize the biophysical forces acting in the dynamic experimental setup.

Once the geometries and physical parameters of the system were established (see STAR Methods), we simulated fluid flow within the chamber and analyzed the resulting streamlines to calculate shear stress on the scaffold surfaces. We considered both the mean and median values because the mean is heavily influenced by peak values that occur in less densely represented regions of the mesh. The mean shear stress on a 5 $\times$ 8-mm scaffold in LB1 at a flow rate of 100 μL/min was 9.03 $\times$ 10^{-10} Pa, while in LB2 at 200 μL/min it was 6.09 $\times$ 10^{-8} Pa (Figures 2BI and 2BII).

We next established the disease model, in which CLL cells were recirculated through the dynamic system comprising the peristaltic pump, reservoir, and LB2 bioreactor. The leukemic cell suspension was introduced into the reservoir and circulated at a flow rate of 100 μL/min, and the wall shear stress acting on the scaffold within the LB2 bioreactor was simulated and analyzed. We detected high variability in the mean shear stress between the scaffold walls, with a standard deviation of the same order of magnitude as the mean. Consequently, we assessed shear stress separately on the upper, lower, and lateral walls of the scaffold (Figure 2C).

To evaluate cell trajectories and distribution, we performed nanoparticle tracking. In the context of the LB2 chamber in the “niche” configuration, with the scaffold positioned in the upper compartment, we saw the cells gradually reach and infiltrate the scaffold over time during recirculation (Video S1). Notably, not all circulating cells were directly conveyed on the scaffold; their entry was not physically constrained but seemed to be influenced by biochemical signaling from the microenvironment.

Dynamic stimulation enhances physiological tissue-like organization

With the dynamic configuration established, we next cultured the 3D tissues under both static and dynamic conditions to compare cell behavior. We processed the seeded scaffolds as actual tissues for histological and immunofluorescence analyses. Samples were fixed, embedded, and sectioned in their entirety to evaluate cell organization and functional marker expression at different scaffold depths.

Macroscopic inspection of the samples revealed clear differences between static and dynamic conditions: the dynamically cultured scaffolds were more compact and less transparent (Figure 3A). This observation was confirmed by hematoxylin and eosin staining (Figure 3B), which showed that dynamic stimulation promoted a more physiological cell morphology, increased cell-cell interactions, and a more complex tissue-like organization in both BM and LN constructs. Indeed, in dynamic culture, cells likely benefit from both the convective transport of oxygen, nutrients, and growth factors and the physical forces present in the system.[35]

We further analyzed the structural organization, initially focusing on cell nuclei, as nuclear morphology and mechanics are tightly linked to cytoskeletal dynamics, sensing and transducing mechanical stimuli.[41] Immunofluorescence staining showed that nuclei of stromal cells in both BM and LN scaffolds were larger and more elongated under dynamic conditions, in contrast to the smaller, rounder nuclei observed in static culture (Figure 3C).

CellPress
OPEN ACCESS

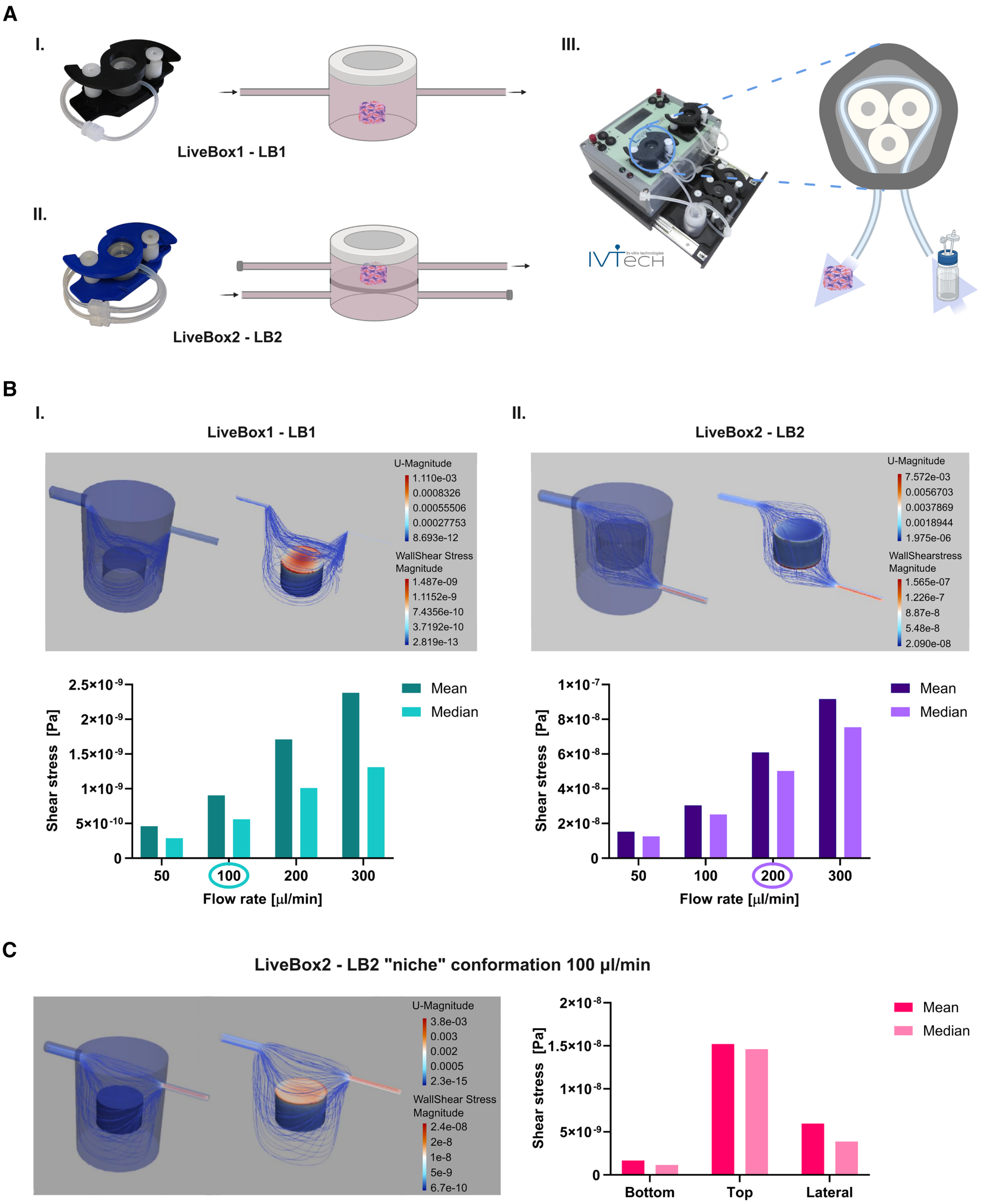

Figure 2. CFD simulation in IVTech dynamic system

In all the CFD images, the flow lines are characterized by a colorimetric map defining the intensity of the sliding speed.

(A) Real pictures of the main components of the dynamic system: (I) single chamber bioreactor, LiveBox1 (LB1); (II) double chamber bioreactor, LiveBox2 (LB2); (III) peristaltic pump, LiveFlow. The bioreactors and the closed-loop fluidic circuit are also graphically represented.

(B) Flow lines in the LB1 (I) and LB2 (II) configurations. The colorimetric maps in images represent the laminar flow rate and the shear stress magnitude. The histogram shows mean and median values of the shear stress correlated with flow speed in both bioreactors.

(C) LiveBox2 “niche” conformation for CLL cells recirculation. (Left) Flow lines representation in the “niche” conformation. (Right) Shear stress mean and median values calculated at 100 µL/min on the top, bottom, and lateral surfaces of the scaffold.

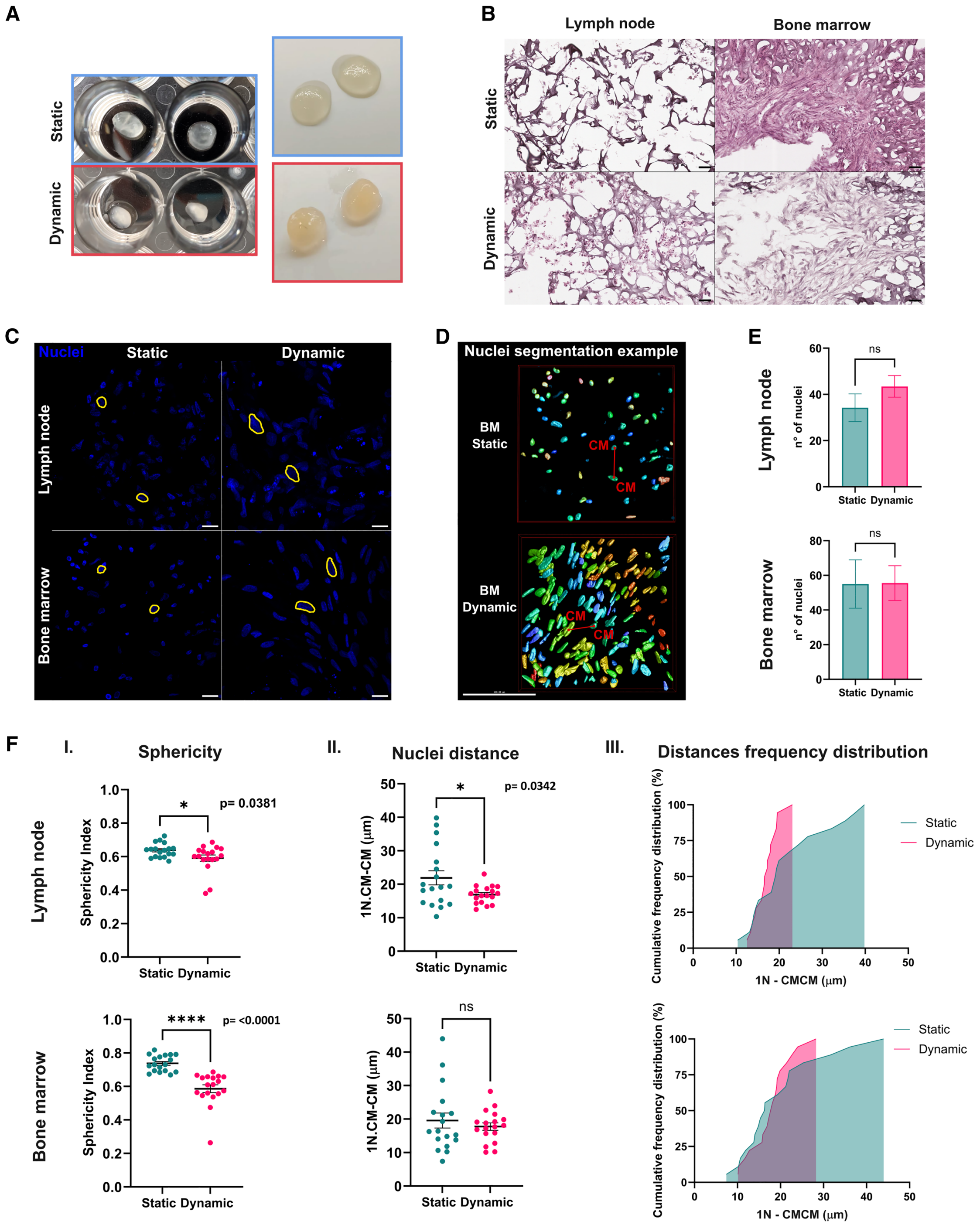

Figure 3. Static vs. dynamic: Morphometric analysis of nuclei
(A) Real images of samples cultured in static and dynamic conditions for 15 days.
(B) Hematoxylin and eosin staining of static and dynamic slices from LN and BM scaffolds. Images were acquired using a 40× objective lens. Scale bar, 50 µm.
(C) Confocal microscopy z stack nuclei reconstruction of both microenvironments, cultured in static and dynamic conditions. z Stacks were acquired using a 60× objective lens. Scale bars, 20 µm.
(D) Example of nuclei segmentation with Huygens software on hMSC scaffolds. CM = center of mass. Scale bar, 100 µm.
(E) Number of nuclei obtained counting the number of total objects segmented from confocal z stack reconstructed images.

(legend continued on next page)

To assess whether cells under perfusion behave more similarly to those in tissue, we performed morphometric[42] and distribution analyses[43] of nuclei from 3D-reconstructed confocal images obtained from different scaffold regions (Figure 3D). Nuclei counts revealed no significant differences between static and dynamic conditions, indicating that the observed differences in organization were not due to variations in cell number (Figure 3E). Nuclei under dynamic stimulation, however, exhibited significantly lower sphericity, suggesting increased elongation in response to mechanical forces, consistent with more tissue-like cell morphology (Figure 3FI).[41,44]

To evaluate nuclear spatial distribution, we measured the minimum distance between each nucleus and its nearest neighbor in 3D-reconstructed images from various scaffold depths (Figure 3FII). Overall, cells cultured under dynamic conditions showed reduced internuclear distances. Moreover, under dynamic culture, nuclear distances converged within a narrower range (10–28 μm) (Figure 3FIII), consistent with the range observed in various *in vivo* tissue types (4–30 μm).[43] These observations suggest that dynamic stimulation enhances the physiological behavior of cells in 3D culture, leading to tissue-like morphology and a more homogeneous distribution within the scaffold.

Perfused 3D tissues exhibit a higher tissue-like complexity

The ECM is a key non-cellular component that supports cell spreading, orchestrates tissue organization, and influences cell fate.[45,46] We thus assessed the presence of functional specific markers and the spatial organization of cells and the produced ECM. Immunofluorescence analysis after 15 days of culture revealed that cells cultured under static conditions showed complete disorganization, including an absence of cadherin expression (Figures 4Aa and 5Aa) and disruption of F-actin filaments (Figures 4Ab and 5Ab). By contrast, cells under dynamic conditions displayed intact F-actin filaments, cadherin expression, and more complex intercellular interactions (Figures 4Ac, 4Ad, 5Ac, and 5Ad). F-actin is closely associated with cadherin adhesion proteins, forming a mechanosensory complex that responds to exogenous forces such as shear stress and matrix stiffness. The cadherin-actin mechanical linkage regulates junction strength and cell-cell interactions, which are essential for maintaining tissue homeostasis.[47,48] These findings suggest that dynamic culture supports cellular equilibrium and organization within the 3D tissue.

Cells exposed to flow stimulation seemed to align both with the scaffold architecture (Figure 4A, yellow circles) and the flow direction (Figures 4A and 5A, white arrows), indicating their ability to sense 3D topography and shear forces.[35] In the LN model, structural and organizational differences in podoplanin-positive ($PDPN^+$) fibroblasts (HLF) and $CD31^+$ endothelial cells (HUVEC) were especially highlighted through immunofluorescence staining (Figure 4A). RT-qPCR analysis confirmed that dynamic conditions upregulated gene expression of PDPN and CD31 (Figure 4B). To quantify cell-type contributions, we dissociated the 3D LN scaffolds and performed flow cytometry. After 15 days, both static and dynamic cultures retained the initial HLF:HUVEC seeding ratio of 1:2 (Figure 4C).

In the BM model, dynamic stimulation led to the expression of both early osteogenic (alkaline phosphatase [ALP]) and endothelial (CD31, collagen IV) markers, as shown by immunofluorescence and RT-qPCR analysis (Figures 5B and 5C).[49–51] These findings suggest that hMSCs respond to a combination of biochemical and mechanical cues from the microenvironment—including shear stress and matrix composition—by directing their differentiation accordingly.[52,53]

The 3D tissues cultured under fluid flow displayed greater ECM maturation, organization, and complexity compared to those maintained in static conditions. In both LN and BM models, cells produced abundant ECM proteins, including fibronectin, laminins, hyaluronic acid, and collagen (Figures 4D and 5D), which are involved in migration, cytoskeletal remodeling, adhesion, proliferation, and differentiation.[49,54–56] Immunofluorescence analysis (Figures 4A and 4D) showed that lymphatic fibroblasts under dynamic flow formed stellate cell-cell contacts and a highly organized ECM mesh that resembled the fibroblastic reticular cell (FRC) network found in LNs.[57,58] Dynamic culture also promoted the formation of $CD31^+$ luminal-like structures aligned with the scaffold pore network (Figures 4Ac and 4Ad, yellow circles). These structures were surrounded by collagen IV[59] and expressed the tight junction protein VE-cadherin (Figure 4Db), which, together with CD31, forms a mechanosensory complex that regulates vascular remodeling and homeostasis in response to shear stress (Video S2).[60,61]

These results demonstrate that both the 3D architecture and dynamic stimulation of the culture system influence cellular behavior. Specifically, dynamic perfusion enhanced cell motility, adhesion, morphological adaptation, ECM protein production, and the development of functional structures such as the FRC-like network and vascular elements in our LN model.

Dynamic stimulation upregulates mechanotransduction and mechanosensory-related genes

We next examined the expression of genes involved in mechanoregulation between static and dynamic conditions. Specifically, we performed RT-qPCR to evaluate lamin A (LMNA), fascin 1 (FSCN1), protein tyrosine kinase 2 (PTK2), intercellular adhesion molecule 1 (ICAM1), and vascular cell adhesion molecule 1 (VCAM1) expression. LMNA, FSCN1, and PTK2 are genes involved in the mechanosensory and mechanotransduction complexes regulating various cell activities correlated with structural integrity, nuclear stability,[41] adhesion, motility,[62] and cytoskeletal remodeling.[49,63] ICAM1 and VCAM1 are involved in leukocyte adhesion to stromal and endothelial cells and are

(F) Analysis of sphericity (I) and distances (II, III) of nuclei performed on confocal reconstructed images. Sphericity is defined by values going from 0 to 1, where 1 identifies a perfect sphere and 0 defines a more elongated object. The second column displays values of minimum distances between one nucleus and its nearest neighbor. The last column shows the same results of distances in a cumulative relative distribution frequency. All data are represented by mean ± SEM. Unpaired t test with Welch's correction was performed ($p < 0.05$). The analysis was carried out on three different depth areas in each sample, two slices for each depth. For every condition (i.e., LN static and dynamic, BM static and dynamic), three biological replicates have been analyzed.

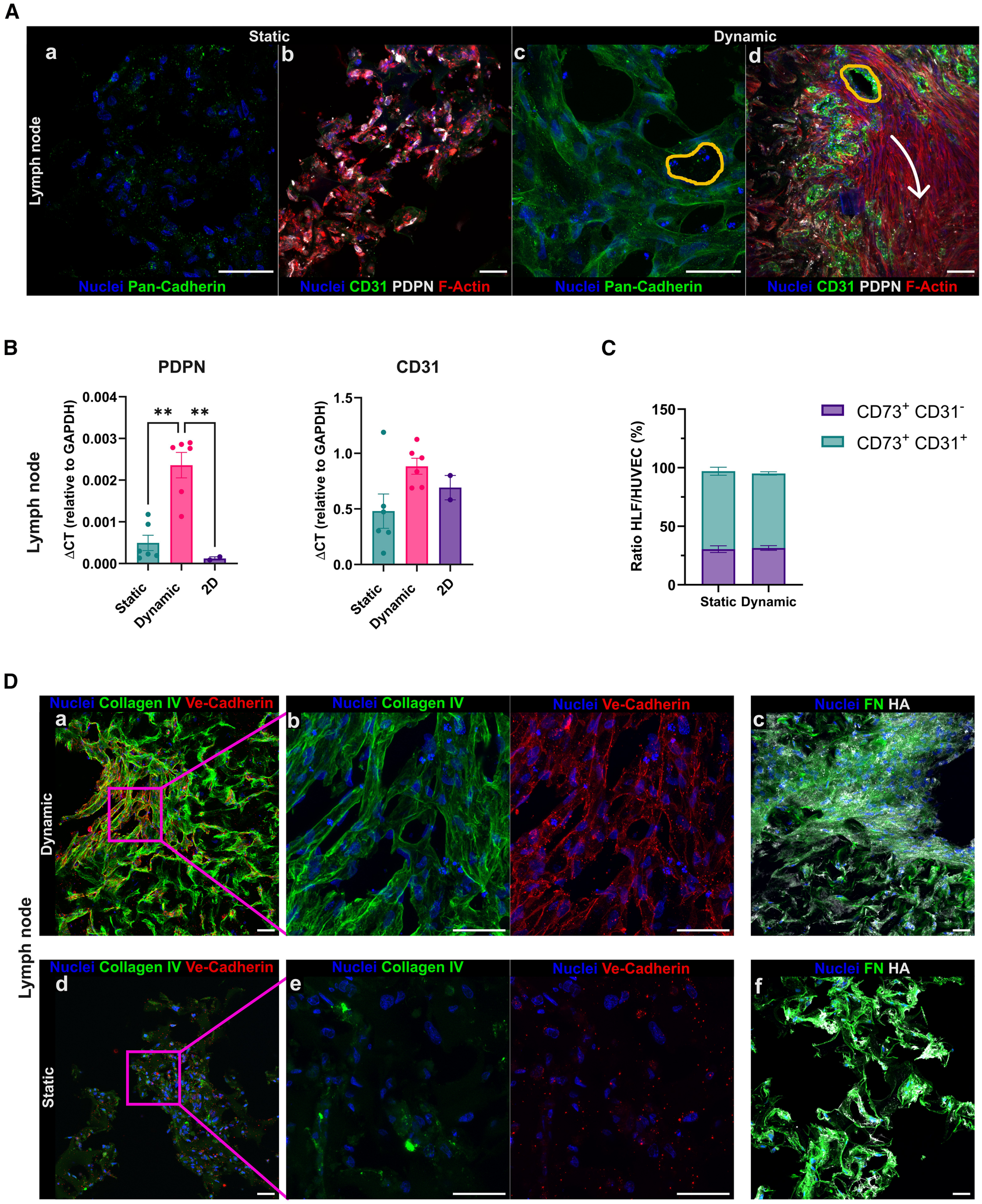

Figure 4. Static vs. dynamic: The lymph node

(A) Expression comparison of specific functional markers between static and dynamic scaffolds. White arrow indicates the direction through which the cells organize following the direction of the flow. Yellow circles indicate the material pores around which endothelial cells position and organize. z Stacks were acquired using a 30× (a, c) and a 60× (b, d) objective lens.

(B) RT-qPCR expression analysis of PDPN and CD31 in the LN model. The analysis was made on static 3D culture, dynamic 3D culture, and 2D culture. The data have been normalized on the glyceraldehyde 3-phosphate dehydrogenase (GAPDH) housekeeping gene. All data are represented by mean ± SEM. Tukey's multiple comparison test was performed; $p < 0.05$; $n = 6$ for 3D static and dynamic conditions, $n = 2$ for 2D condition.

(C) Flow cytometry analysis of the ratio between HLF and HUVEC cells after 15 days of maturation, showing the contribution in terms of percentage of the two cell types in the LN model. HLF cells are $CD73^{+}/CD31^{-}$, and HUVEC cells are $CD73^{+}/CD31^{+}$. Data are represented by mean ± SEM.

(D) Comparison of ECM protein deposition between static and dynamic scaffolds of the LN model. z Stacks were acquired using a 20× (a, c, d, f) and a 60× (b, e) objective lens. Scale bars, 50 μm.

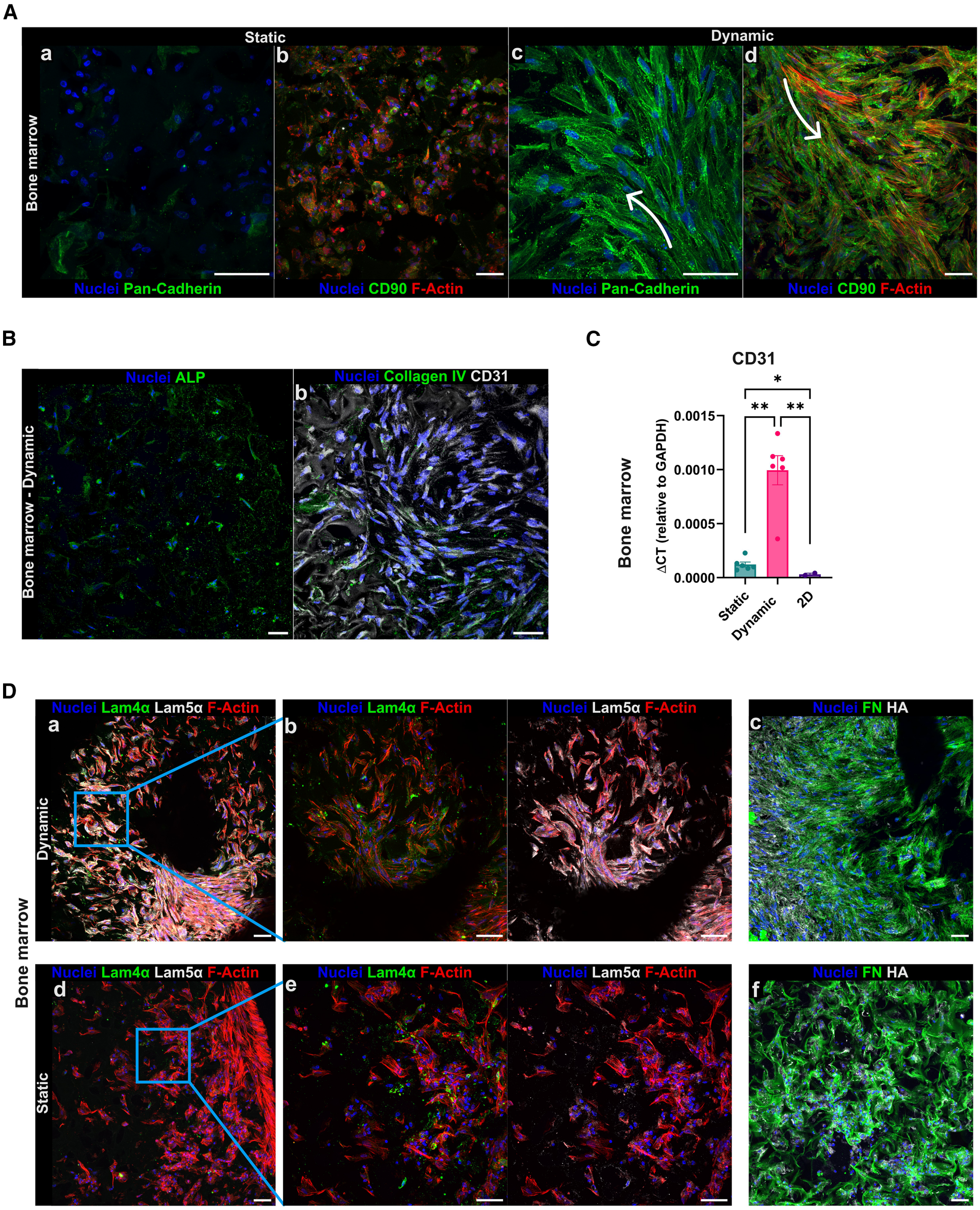

Figure 5. Static vs. dynamic: The bone marrow

(A) Expression comparison of specific functional markers between static and dynamic scaffolds. White arrows indicate the direction through which the cells organize following the direction of the flow. Yellow circles indicate the material pores around which endothelial cells position and organize. z Stacks were acquired using a 30× (a, c) and a 60× (b, d) objective lens. Scale bars, 50 μm.

(B) Immunofluorescence staining for alkaline phosphatase (ALP), CD31, and collagen IV in the BM model in dynamic condition. z Stacks were acquired using a 20× (a) and a 30× (b) objective lens. Scale bars, 50 μm.

(C) RT-qPCR expression analysis of CD31 in the BM model in static 3D culture, dynamic 3D culture, and 2D culture. The data have been normalized on the GAPDH housekeeping gene. All data are represented by mean ± SEM. Tukey's multiple comparison test was performed; $p < 0.05$; $n = 6$ for 3D static and dynamic conditions, $n = 2$ for 2D condition.

(D) Comparison of ECM proteins deposition between static and dynamic scaffolds of the BM model. z Stacks were acquired using a 20× (a, c, d, f) and a 30× (b, e) objective lens. Scale bars, 50 μm.

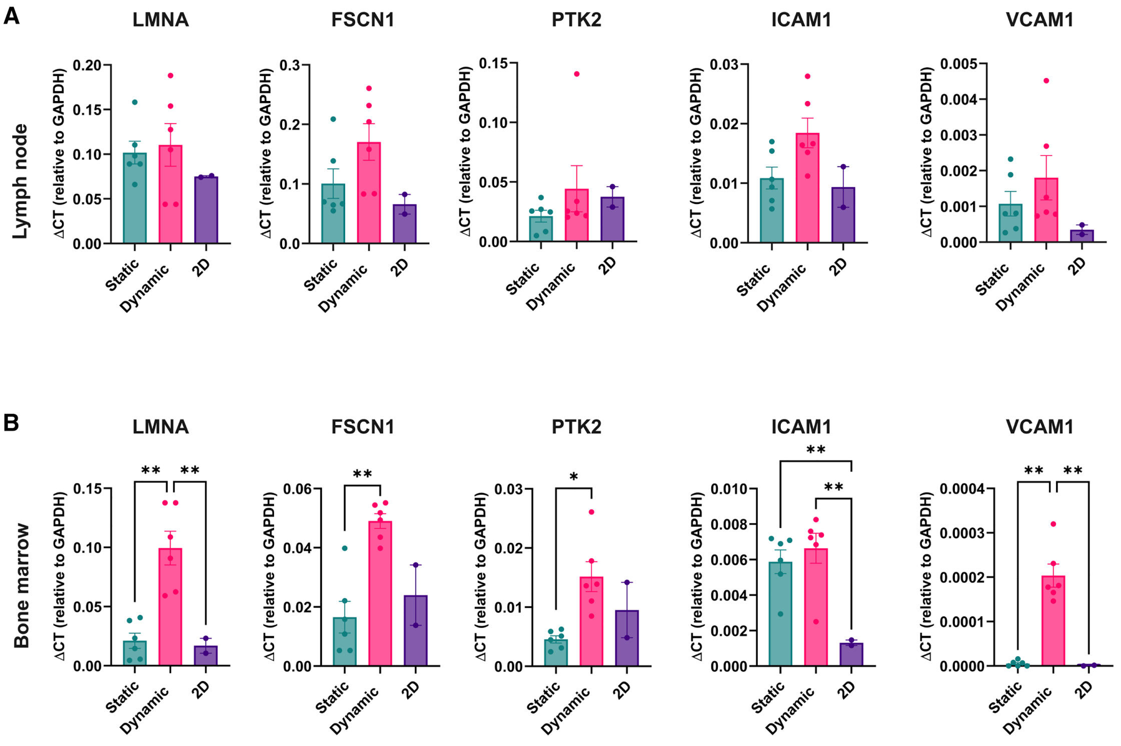

Figure 6. RT-qPCR gene expression analysis

RT-qPCR expression analysis of LMNA, FSCN1, PTK2, ICAM1, and VCAM1 in both LN (A) and BM (B). The analysis was made on three conditions: static 3D culture, dynamic 3D culture, and 2D culture. The data have been normalized on the GAPDH housekeeping gene. All data are represented by mean ± SEM. Tukey's multiple comparison test; $p < 0.05$; $n = 6$ for 3D static and dynamic conditions and $n = 2$ for 2D condition.

critical for transendothelial migration.[64,65] In both microenvironments, we observed a general trend toward increased LMNA and FSCN1 expression in 3D cultures compared with 2D, with statistically significant differences in the BM samples (Figures 6A and 6B). In the BM model specifically, dynamic conditions further enhanced the expression of both genes compared to static culture. PTK2 was also significantly upregulated under dynamic conditions, further supporting the cellular responsiveness to biomechanical stimuli within 3D and perfused environments. Scaffolds cultured under dynamic conditions showed significantly elevated expression of ICAM1 and VCAM1, further supporting the physiological relevance of our model. These findings indicate that the dynamic 3D system is not only compatible with the recirculation of leukemic cells but also suitable for studying mechanisms of cell migration, homing, and dissemination in tissue-like environments (Figures 6A and 6B).

Recirculating CLL cells home to 3D microenvironments and alter functional marker expression

Like healthy leukocytes, leukemic cells respond to both biochemical and mechanical cues from surrounding tissues and are influenced by shear stress in the bloodstream, which drives cytoskeletal reorganization and deformation necessary for transmigration through the endothelium.[66] To model this behavior, we developed a disease model using our dynamic system (Figure 2), incorporating a single microenvironment (BM or LN) in which the CLL cell line MEC1[67] was resuspended and recirculated under controlled flow. The dynamic setup used here differs from those applied during stromal tissue maturation. Specifically, we used the double-chamber LB2 bioreactor with the scaffold placed in the upper chamber; however, unlike the LN maturation setting, the flow was tangential only in the upper chamber. This configuration prevented CLL cells from becoming trapped beneath the membrane dividing the two chambers, allowing them to partially flow through and interact with the scaffold (Video S1 and scheme in Figure 7A). Importantly, CLL cells were not physically forced into the scaffold by the flow but instead seemed to be guided by biochemical signals produced by the stromal cells. To minimize confounding effects from stromal cell-derived growth factors, we used the standard MEC1 culture medium during recirculation, thereby reducing system variability. Using flow cytometry, we first assessed the viability of MEC1 cells circulating in the system without a scaffold, under varying concentrations and flow rates. Cell viability remained high across all tested conditions (Figure S2). Immunofluorescence imaging (Figure 7A) confirmed that CD45^{+} MEC1 cells were capable of homing into both BM and LN microenvironments and interacting with stromal and endothelial cells in the LN model. Specifically, we documented CD45^{+} MEC1 cells squeezing between stromal and endothelial cells, suggesting active migration (Figure 7Ab, yellow arrow). We then analyzed circulating MEC1 cells for the expression of a panel of clinically relevant prognostic markers, aiming to assess whether the model can capture CLL behavior in response to environmental stimuli or therapeutic modulation. Although MEC1 cells inherently

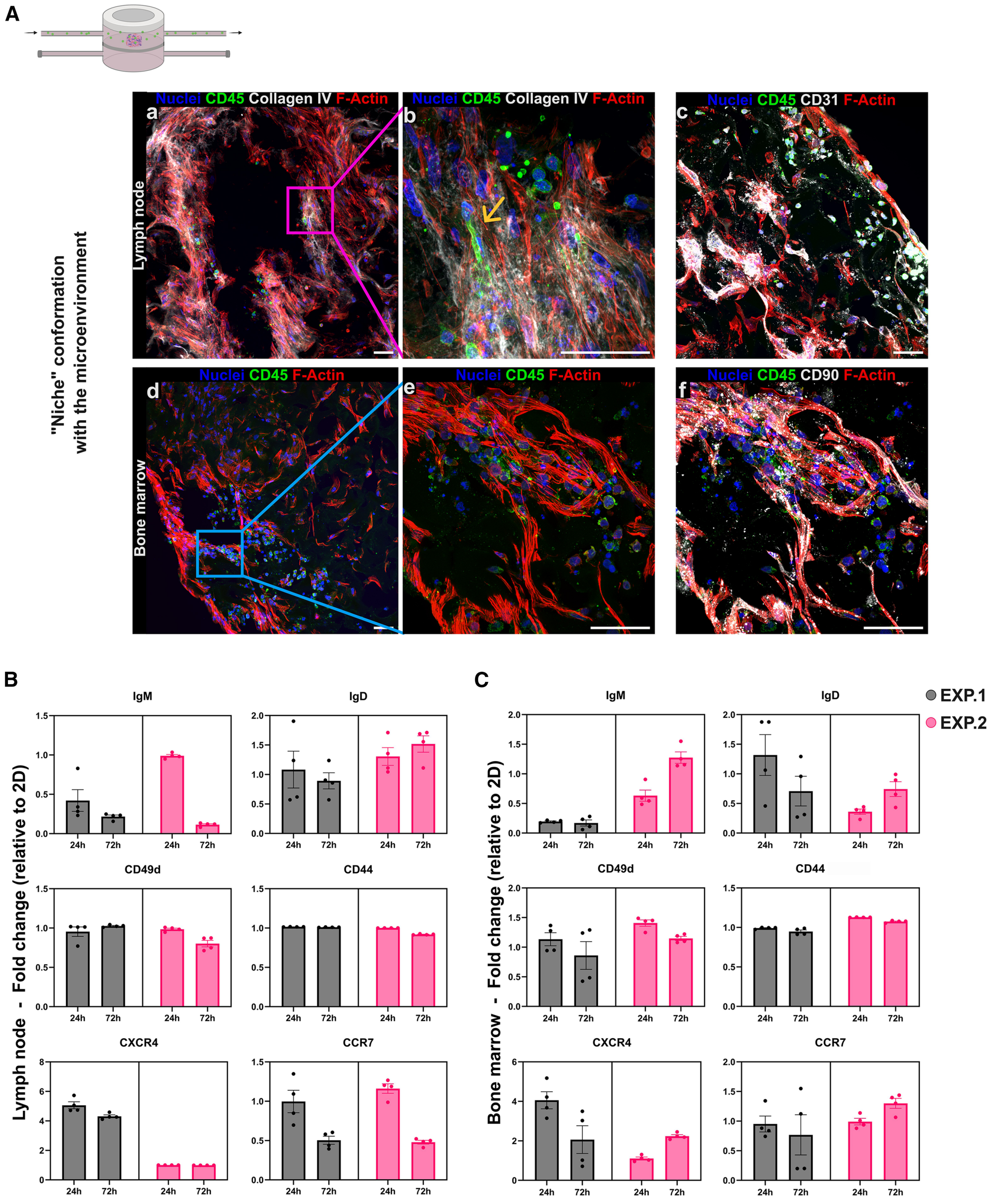

Figure 7. The disease model

(A) Recirculation of MEC1 CLL cells in the "niche" conformation with the microenvironment. Immunofluorescence images showing $CD45^+$ (green) MEC1 cells in the LN and BM microenvironments. Z Stacks were acquired using a 20× (a, d), a 30× (c), a 60× (e, f), and a 60× with 1.5 zoom (b) objective lens. Scale bars, 50 μm.

(B) Flow cytometry analysis of CLL-specific markers on circulating cells in the LN model.

(C) Flow cytometry analysis of CLL-specific markers on circulating cells in the BM model. For all the markers, data are represented by fold change relative to the 2D condition. All data are represented by mean ± SEM. For each model (LN and BM) two independent experiments (EXP.1 in black, EXP.2 in pink), with four replicates each were performed.

express high baseline levels of various CLL markers (which can vary in expression among patient samples), we detected significant changes in marker expression in recirculating cells, including B cell receptor (BCR) isoforms immunoglobulin D (IgD) and IgM[68]; cell adhesion, activation, migration, and retention markers such as as CD23,[69] CD62L,[70] CD69,[71] VLA-4 (CD49d/CD29),[72] CD44,[73] CD38,[74] CD40, and CD80[75] and homing chemokine receptors CXCR4[76] and CCR7[77] (Figures 7B, 7C, and S2; Table S1). Figures 7B and 7C show the flow cytometry data for two independent experiments (with four biological replicates each) of MEC1 cells circulation in the two microenvironments. We achieved a strong reproducibility among the biological replicates of the single experiment. For most of the examined markers, we were able to observe maintenance of the trend variation compared to the 2D condition. However, we observed a variable expression of the markers among the two experiments, which is due to the basal variability of the MEC1 cell line in culture, the same that we expect to find with primary cells isolated from patients. The most affected markers appeared to be homing receptors, which may depend on the snapshot in which we recover recirculating CLL cells: exiting or entering the microenvironment. To this end, we aimed to set up an effective disaggregation protocol to recover and analyze all CLL cells inside the microenvironments. This demonstrated that we were able to generate reproducible 3D BM and LN scaffolds in which to also study the modulation of relevant markers in variable conditions.

DISCUSSION

Bioengineering complex tissues such as lymphoid organs is increasingly important not only for studying hematologic cancers but also for understanding physiological processes like hematopoiesis and immune responses. Over the past several decades, 3D *in vitro* modeling and tissue engineering have garnered increasing interest in the scientific community because they offer innovative approaches to recreate complex microenvironments for studying pathophysiological processes *ex vivo*.[11] In the context of hematological malignancies, the interactions between neoplastic cells and their surrounding microenvironment are particularly critical.[13] CLL, in particular, is a heterogeneous blood cancer characterized by substantial intra- and inter-clonal variability, contributing to high relapse rates. CLL cell activation occurs primarily within lymphoid tissues, where biochemical and mechanical cues promote disease progression.[16,18] In this study, we optimized a 3D dynamic system that mimics LN and BM microenvironments to recirculate CLL cells and investigate mechanisms of dissemination and homing *ex vivo*. Our model is based on the maturation of macroscale lymphoid tissue-like scaffolds within bioreactors, integrated into a milli-fluidic dynamic culture system.

Many existing 3D culture models rely on widely used self-assembly techniques, where cancer cells form spheroids or organoids.[11] To overcome the challenge of blood cells not forming 3D aggregates amenable to downstream analysis, we used a scaffold-based seeding strategy that facilitates tissue-like samples handling and enables integration of microenvironmental cues and mechanical stimulation to better mimic *in vivo* conditions. Moving beyond conventional organ-on-a-chip[29] and microfluidic platforms, we scaled up our model using a milli-fluidic system able to accommodate larger tissue constructs and recapitulate recirculation of leukemic cells through different tissues.[9]

Given the purpose of the study, we began by characterizing a biocompatible gelatin-based biomaterial, Spongostan. This matrix features a heterogeneous internal topography and mechanical properties that support the viability, proliferation, organization, and differentiation of the cells used.[32] This structure facilitates cell migration and elongation throughout the scaffold, promoting robust cell-cell interactions. Its gelatin-based composition and tunable stiffness further enhance cell viability, proliferation, and differentiation.

As cells are initially seeded onto the dry scaffold and then cultured in the same medium-soaked matrix, we evaluated the Young's modulus in both conditions: 11 kPa when dry and 1 kPa when wet. To contextualize our findings, we considered multiple studies assessing lymphoid tissue stiffness and confirmed that our measurements for Spongostan fall within the range reported for soft tissues: 0.25–24.7 kPa for BM and 2.30–7.81 kPa for SLO.[38–40] The selection of this material, with its specific stiffness profile, was also made with a view to developing the BM model further, by introducing differentiation stimuli to mimic both vascular and endosteal niches. Notably, Zhang et al. reported that softer scaffolds (0.66 ± 0.08 kPa) not only promoted MSC proliferation but also accelerated osteogenic differentiation and inorganic matrix deposition.[78]

Once the suitability of the biomaterial for supporting the selected cell types was confirmed, we cultured the scaffolds under both static and dynamic conditions. The dynamic setting used for tissue maturation was selected following multiple optimization experiments and was subsequently characterized and validated through CFD analysis. This approach provided insights into the physical forces acting within the bioreactors, thereby improving the reproducibility of the model generation protocol. The adoption of a perfused culture configuration was motivated by the need to recapitulate the physiological trafficking of CLL cells through circulation between tissues. This choice was further reinforced by the observed advantages of perfusion on scaffold maturation and tissue development.

One of the first noticeable differences between static and dynamic cultures was the macroscopic appearance of the constructs: perfused scaffolds appeared more compact and elastic to the touch. At the microscopic level, pronounced differences in nuclear morphology, cell distribution, and organization were also evident. In static cultures, cells failed to populate regions beyond the surface scaffold. By contrast, medium recirculation in the dynamic system not only enhanced the delivery of oxygen and nutrients and the removal of waste but also introduced mechanical cues that stimulated cell migration and organization.[9]

Although cell viability was maintained in static conditions for up to 15 days, cells in the dynamic system exhibited a more elongated morphology and were distributed more uniformly throughout the scaffold. Cells cultured under dynamic stimulation also displayed significantly lower nuclear sphericity, a metric indicating deviation from a perfect sphere if sphericity = 1,[42] with shorter minimum internuclear distances from one to another. This finding is indicative of more elongated cells with a compact cell arrangement, reflective of a tissue-like organization.[43] Importantly, the number of nuclei did not differ

CellPress
OPEN ACCESS

significantly between the two conditions in either the LN or BM model, indicating that the enhanced organization in the dynamic cultures was not due to higher cell numbers, but to a direct consequence of perfusion. Notably, cells under perfusion tended to cluster centrally within the scaffold, spreading outward to organize and build the ECM protein net. This process is time intensive, and although the scaffold was fully populated with cells after 15 days, it had not yet achieved the complete complexity of a mature tissue-like architecture. Given the 3D nature of the construct and the heterogeneous cell distribution, we performed morphometric and distance analyses on multiple scaffold sections obtained from different depths to account for regional variability in cell density across biological replicates. In the perfused samples, cells were more homogeneously distributed, more elongated, and organized in a tissue-like manner.[43] Looking ahead, we aim to optimize the ratio of matrix volume to seeded cell number to accelerate tissue maturation and achieve more uniform tissue coverage in a shorter time frame.

Through immunofluorescence staining, we observed differences in cell morphology and organization, along with the expression and localization of key cytoskeletal regulators and functional markers. Static samples displayed disrupted actin filaments and an absence of cadherin expression—both essential for maintaining tissue homeostasis and intercellular adhesion.[47,48] Furthermore, RT-qPCR analysis revealed higher expression of genes encoding mechanotransduction proteins—FSCN1, LMNA, and PTK2—in dynamic cultures. These genes are involved in actomyosin complex activity, which governs cytoskeletal rearrangements, migration, and both cell-cell and cell-ECM adhesion.[35,49] Cells in the dynamic condition sensed the 3D structure and fluid movement within the scaffold, organizing accordingly and producing key ECM components such as fibronectin, hyaluronic acid, laminins, and collagen. This complex ECM network is essential for recreating the intricate architecture of LN and BM.[55–58] The microarchitecture of human LN is tightly regulated by FRCs, whose spatial organization defines compartmentalized niches and shapes both innate and adaptive immune responses.[58] In our 3D LN model, we observed expression of PDPN, a specific marker of FRCs, as well as $CD31^+$ endothelial structures accompanied by collagen IV deposition. Gene expression analysis further confirmed the trend toward increased PDPN and CD31 expression under dynamic conditions. These $CD31^+$ luminal-like structures aligned along the scaffold pore network and exhibited a cobblestone-like endothelial arrangement marked by VE-cadherin expression.[60]

hMSCs are classically recognized for their adipogenic, chondrogenic, and osteogenic differentiation potential, and they also exhibit plasticity toward other lineages, including neural, endothelial lineages.[79] As in the LN model, dynamic conditions enhanced hMSC stimulation in the BM model through both osteogenic and endothelial differentiation.[23,49] Future iterations of the model will thus include specific differentiation factors in the culture medium. Such endothelialization of the 3D scaffolds is not only critical for tissue homeostasis but also plays a role in tumor-stroma interactions within the disease model. Indeed, we saw that dynamic 3D cultures of both LN and BM expressed higher levels of ICAM1 and VCAM1, encoding adhesion molecules involved in leukocyte transendothelial migration.[64,65]

Following successful maturation of the lymphoid-like tissues, we finally developed a disease model using the MEC1 CLL cell line. These leukemic cells were able to home to both 3D microenvironments and establish interactions with stromal and endothelial components, including active migration through the stromal network. Interestingly, through flow cytometric analysis, we observed distinct differences not only between MEC1 cells cultured in 3D dynamic versus 2D static conditions but also between cells recirculated within the BM versus LN microenvironments. In particular, modulation of BCR isoforms IgM and IgD is of clinical relevance, given their prognostic value and the central role of BCR-targeting therapies, such as BTK inhibitors (e.g., ibrutinib).[68] The expression of VLA-4 (CD49d/CD29)—a key prognostic marker involved in adhesion via binding to VCAM1 and fibronectin, both produced by our 3D models—was also regulated.[72] Additionally, we observed changes in CD44 expression, another critical marker associated with tumor progression and microenvironmental interaction, which binds hyaluronic acid, an ECM component present in both models.[76]

Limitations of the study

With this study, we assessed the feasibility and the importance of this platform, but the study has some limitations. In the future we will address the previously mentioned issue of the inability of cells to fill the entire scaffold in relatively short cultures, playing on the ratio between cell number and scaffold volume. We also aim to increase the complexity of both tissue models by incorporating additional microenvironmental components getting closer to the *in vivo* tissues. Another important future experiment would be to compare the cellular morphologies and organization of our model to human BM and LN samples to better assess physiological relevance. Similarly, in this work, we used a CLL cell line, which was useful to set up the platform, but we could not recapitulate patients' cell behavior and expression of clinically relevant markers. Therefore, in the future we will substitute the cell line with patients' cells. Moving forward, we expect our model to set the stage for a perfused multi-organ platform in which multiple compartments are interconnected. In this way, we will allow for comprehensive, physiologically relevant modeling of CLL progression and treatment responses.

In conclusion, we successfully established a 3D perfused model that mimics lymphoid microenvironments and provides a functional platform to study pathological mechanisms in CLL. The scale-up of the disease model by introducing bloodstream-like recirculation of CLL cells into and out of the microenvironments, is now feasible. Such work will allow for more accurate studies of leukemic cell mobilization, dissemination, and response to targeted therapies.

RESOURCE AVAILABILITY

Lead contact

Further information and requests for resources and reagents should be directed to and will be fulfilled by the lead contact, Cristina Scielzo (scielzo.cristina@hsr.it).

Materials availability

This study did not generate any new unique reagents.

Data and code availability

- Data generated by this study is available from the lead contact upon request.
- This study does not report original code.
- Any additional information required to analyze the data reported in this work paper is available from the lead contact upon request.

ACKNOWLEDGMENTS

C.S. acknowledges financial support from the The European Hematology Association (EHA) - Advanced Research Grant 2020; Alternatives Research & Development Foundation (ARDF) - Grant 2022; and the Associazione Italiana per la Ricerca sul Cancro (AIRC) under IG 2018 – ID, 21332, and IG 2023 - ID28750 and the Special Program on Metastatic Disease – 5 per mille no. 2119. We thank the alembic and fractal facilities for their support. Schematics representations in Figures 1A, 1B, 1E, 1F, 2A, 7A, S2A, and the graphical abstract were created with BioRender.com. We thank Dr. Jessica Tamanini for the editing work on the manuscript.

AUTHOR CONTRIBUTIONS

D.B. and C.S. wrote the article. D.B., F.B., F.S., D.R., M.S., M.P., B.V., and R.P. performed the experiments. D.B. and D.R. performed the analysis. S.F. supervised the mechanical analysis of the biomaterial. V.B. supervised the confocal microscopy analysis. C.S., F.M., P.G., and D.B. revised the article. C.S. and F. M. supervised the activity.

DECLARATION OF INTERESTS

The authors declare no competing interests.

STAR★METHODS

Detailed methods are provided in the online version of this paper and include the following:

- KEY RESOURCES TABLE
- EXPERIMENTAL MODEL AND STUDY PARTICIPANT DETAILS
  - HLF cell culture
  - HUVEC cell culture
  - hMSC cell culture
  - MEC1 cell culture
- METHOD DETAILS
  - Biomaterial characterization: Spongostan
  - Cell transduction
  - 3D scaffold generation
  - Live/dead assay
  - Alamar Blue assay
  - Dynamic system
  - CLL cell line recirculation in the dynamic system
  - Computational fluid dynamic study (CFD)
  - Scaffold processing for imaging analysis
  - Histochemistry
  - Immunofluorescence analyses
  - Morphological confocal analysis
  - RNA extraction and real-time PCR
  - Scaffold processing for flow cytometry
  - Flow cytometric analyses
- QUANTIFICATION AND STATISTICAL ANALYSIS

SUPPLEMENTAL INFORMATION

Supplemental information can be found online at https://doi.org/10.1016/j.crmeth.2025.101105.

Received: January 2, 2025
Revised: May 5, 2025
Accepted: June 17, 2025
Published: July 11, 2025

REFERENCES

1. Jensen, C., and Teng, Y. (2020). Is It Time to Start Transitioning From 2D to 3D Cell Culture? Front. Mol. Biosci. *7*, 33. https://doi.org/10.3389/fmolb.2020.00033.
2. Flecknell, P. (2002). Replacement, Reduction and Refinement. ALTEX *19*, 73–78. https://altex.org/index.php/altex/issue/view/66?iid=65&aid=5.
3. Van Norman, G.A. (2019). Limitations of Animal Studies for Predicting Toxicity in Clinical Trials: Is it Time to Rethink Our Current Approach? JACC Basic Transl Sci *4*, 845–854. https://doi.org/10.1016/j.jacbts.2019.10.008.
4. Pampaloni, F., Reynaud, E.G., and Stelzer, E.H.K. (2007). The third dimension bridges the gap between cell culture and live tissue. Nat. Rev. Mol. Cell Biol. *8*, 839–845. https://doi.org/10.1038/nrm2236.
5. Baker, B.M., and Chen, C.S. (2012). Deconstructing the third dimension-how 3D culture microenvironments alter cellular cues. J. Cell Sci. *125*, 3015–3024. https://doi.org/10.1242/jcs.079509.
6. Brancato, V., Oliveira, J.M., Correlo, V.M., Reis, R.L., and Kundu, S.C. (2020). Could 3D models of cancer enhance drug screening? Biomaterials *232*, 119744. https://doi.org/10.1016/j.biomaterials.2019.119744.
7. Rodrigues, J., Heinrich, M.A., Teixeira, L.M., and Prakash, J. (2021). 3D In Vitro Model (R)evolution: Unveiling Tumor–Stroma Interactions. Trends Cancer *7*, 249–264. https://doi.org/10.1016/j.trecan.2020.10.009.
8. Zemel, A., Rehfeldt, F., Brown, A.E.X., Discher, D.E., and Safran, S.A. (2010). Optimal matrix rigidity for stress-fibre polarization in stem cells. Nat. Phys. *6*, 468–473. https://doi.org/10.1038/nphys1613.
9. Karami, D., Richbourg, N., and Sikavitsas, V. (2019). Dynamic in vitro models for tumor tissue engineering. Cancer Lett. *449*, 178–185. https://doi.org/10.1016/j.canlet.2019.01.043.
10. Rutkowski, J.M., and Swartz, M.A. (2007). A driving force for change: interstitial flow as a morphoregulator. Trends Cell Biol. *17*, 44–50. https://doi.org/10.1016/j.tcb.2006.11.007.
11. Barozzi, D., and Scielzo, C. (2023). Emerging Strategies in 3D Culture Models for Hematological Cancers. Hemasphere *7*, E932. https://doi.org/10.1097/HS9.0000000000000932.
12. Whiteley, A.E., Price, T.T., Cantelli, G., and Sipkins, D.A. (2021). Leukaemia: a model metastatic disease. Nat. Rev. Cancer *21*, 461–475. https://doi.org/10.1038/s41568-021-00355-z.
13. Clara-Trujillo, S., Ferrer, G.G., and Ribelles, J.L.G. (2020). In vitro modeling of non-solid tumors: How far can tissue engineering go? Int. J. Mol. Sci. *21*, 1–31. https://doi.org/10.3390/ijms21165747.
14. Del Giudice, I., Marinelli, M., Wang, J., Bonina, S., Messina, M., Chiaretti, S., Ilari, C., Cafforio, L., Raponi, S., Mauro, F.R., et al. (2016). Inter-and intra-patient clonal and subclonal heterogeneity of chronic lymphocytic leukaemia: Evidences from circulating and lymph nodal compartments. Br. J. Haematol. *172*, 371–383. https://doi.org/10.1111/bjh.13859.
15. Burger, J.A., Ghia, P., Rosenwald, A., and Caligaris-Cappio, F. (2009). The microenvironment in mature B-cell malignancies: A target for new treatment strategies. Blood *114*, 3367–3375. https://doi.org/10.1182/blood-2009-06-225326.
16. Scielzo, C., and Ghia, P. (2020). Modeling the Leukemia Microenviroment In Vitro. Front. Oncol. *10*, 607608. https://doi.org/10.3389/fonc.2020.607608.
17. Haselager, M.V., Kater, A.P., and Eldering, E. (2020). Proliferative Signals in Chronic Lymphocytic Leukemia; What Are We Missing? Front. Oncol. *10*, 592205. https://doi.org/10.3389/fonc.2020.592205.
18. Caligaris-Cappio, F., Bertilaccio, M.T.S., and Scielzo, C. (2014). How the microenvironment wires the natural history of chronic lymphocytic leukemia. Semin. Cancer Biol. *24*, 43–48. https://doi.org/10.1016/j.semcancer.2013.06.010.

CellPress
OPEN ACCESS

19. Primo, D., Scarfò, L., Xochelli, A., Mattsson, M., Ranghetti, P., Belén Espinosa, A., Robles, A., Gorrochategui, J., Martínez-López, J., De La Serna, J., et al. (2018). A novel ex vivo high-throughput assay reveals antiproliferative effects of idelalisib and ibrutinib in chronic lymphocytic leukemia. Oncotarget *9*, 26019–26031.

20. Sbrana, F.V., Pinos, R., Barbaglio, F., Ribezzi, D., Scagnoli, F., Scarfò, L., Redwan, I.N., Martinez, H., Farè, S., Ghia, P., and Scielzo, C. (2021). 3D Bioprinting Allows the Establishment of Long-Term 3D Culture Model for Chronic Lymphocytic Leukemia Cells. Front. Immunol. *12*, 639572. https://doi.org/10.3389/fimmu.2021.639572.

21. Barbaglio, F., Belloni, D., Scarfò, L., Sbrana, F.V., Ponzoni, M., Bongiovanni, L., Pavesi, L., Zambroni, D., Stamatopoulos, K., Caiolfa, V.R., et al. (2021). Three-dimensional co-culture model of chronic lymphocytic leukemia bone marrow microenvironment predicts patient-specific response to mobilizing agents. Haematologica *106*, 2334–2344. https://doi.org/10.3324/haematol.2020.248112.

22. Belloni, D., Ferrarini, M., Ferrero, E., Guzzeloni, V., Barbaglio, F., Ghia, P., and Scielzo, C. (2022). Protocol for generation of 3D bone marrow surrogate microenvironments in a rotary cell culture system. STAR Protoc. *3*, 101601. https://doi.org/10.1016/j.xpro.2022.101601.

23. Fischer, L.J., McIlhenny, S., Tulenko, T., Golesorkhi, N., Zhang, P., Larson, R., Lombardi, J., Shapiro, I., and DiMuzio, P.J. (2009). Endothelial Differentiation of Adipose-Derived Stem Cells: Effects of Endothelial Cell Growth Supplement and Shear Force. J. Surg. Res. *152*, 157–166. https://doi.org/10.1016/j.jss.2008.06.029.

24. Cupedo, T., Stroock, A., and Coles, M. (2012). Application of tissue engineering to the immune system: Development of artificial lymph nodes. Front. Immunol. *3*, 343. https://doi.org/10.3389/fimmu.2012.00343.

25. Di Maggio, N., Piccinini, E., Jaworski, M., Trumpp, A., Wendt, D.J., and Martin, I. (2011). Toward modeling the bone marrow niche using scaffold-based 3D culture systems. Biomaterials *32*, 321–329. https://doi.org/10.1016/j.biomaterials.2010.09.041.

26. Rödling, L., Schwedhelm, I., Kraus, S., Bieback, K., Hansmann, J., and Lee-Thedieck, C. (2017). 3D models of the hematopoietic stem cell niche under steady-state and active conditions. Sci. Rep. *7*. https://doi.org/10.1038/s41598-017-04808-0.

27. Shah, S.B., and Singh, A. (2017). Creating artificial lymphoid tissues to study immunity and hematological malignancies. Curr. Opin. Hematol. *24*, 377–383. https://doi.org/10.1097/MOH.0000000000000356.

28. Di Buduo, C.A., Wray, L.S., Tozzi, L., Malara, A., Chen, Y., Ghezzi, C.E., Smoot, D., Sfara, C., Antonelli, A., Spedden, E., et al. (2015). Programmable 3D silk bone marrow niche for platelet generation ex vivo and modeling of megakaryopoiesis pathologies. Blood *125*, 2254–2264. https://doi.org/10.1182/blood-2014-08.

29. Torisawa, Y.S., Spina, C.S., Mammoto, T., Mammoto, A., Weaver, J.C., Tat, T., Collins, J.J., and Ingber, D.E. (2014). Bone marrow-on-a-chip replicates hematopoietic niche physiology in vitro. Nat. Methods *11*, 663–669. https://doi.org/10.1038/nmeth.2938.

30. Ghoshal, D., Petersen, I., Ringquist, R., Kramer, L., Bhatia, E., Hu, T., Richard, A., Park, R., Corbin, J., Agarwal, S., et al. (2025). Multi-niche human bone marrow on-a-chip for studying the interactions of adoptive CAR-T cell therapies with multiple myeloma. Biomaterials *316*, 123016. https://doi.org/10.1016/j.biomaterials.2024.123016.

31. Tomei, A.A., Siegert, S., Britschgi, M.R., Luther, S.A., and Swartz, M.A. (2009). Fluid Flow Regulates Stromal Cell Organization and CCL21 Expression in a Tissue-Engineered Lymph Node Microenvironment. J. Immunol. *183*, 4273–4283. https://doi.org/10.4049/jimmunol.0900835.

32. Vordemvenne, T., Wähnert, D., Koettnitz, J., Merten, M., Fokin, N., Becker, A., Büker, B., Vogel, A., Kronenberg, D., Stange, R., et al. (2020). Bone Regeneration: A Novel Osteoinductive Function of Spongostan by the Interplay between Its Nano-and Microtopography. Cells *9*, 654. https://doi.org/10.3390/cells9030654.

33. Belloni, D., Heltai, S., Ponzoni, M., Villa, A., Vergani, B., Pecciarini, L., Marcatti, M., Girlanda, S., Tonon, G., Ciceri, F., et al. (2018). Modeling multiple myeloma-bone marrow interactions and response to drugs in a 3d surrogate microenvironment. Haematologica *103*, 707–716. https://doi.org/10.3324/haematol.2017.167486.

34. Swartz, M.A., and Fleury, M.E. (2007). Interstitial flow and its effects in soft tissues. Annu. Rev. Biomed. Eng. *9*, 229–256. https://doi.org/10.1146/annurev.bioeng.9.060906.151850.

35. Juste-Lanas, Y., Hervas-Raluy, S., Manuel García-Aznar, J., González-Loyola, A., Manuel Garc ıa-Aznar, J., and Gonz alez-Loyola, A. (2023). Fluid flow to mimic organ function in 3D in vitro models. APL Bioeng. *7*, 31501. https://doi.org/10.1063/5.0146000.

36. Loh, Q.L., and Choong, C. (2013). Three-dimensional scaffolds for tissue engineering applications: Role of porosity and pore size. Tissue Eng. Part B Rev. *19*, 485–502. https://doi.org/10.1089/ten.teb.2012.0437.

37. Nikolova, M.P., and Chavali, M.S. (2019). Recent advances in biomaterials for 3D scaffolds: A review. Bioact. Mater. *4*, 271–292. https://doi.org/10.1016/j.bioactmat.2019.10.005.

38. Jansen, L.E., Birch, N.P., Schiffman, J.D., Crosby, A.J., and Peyton, S.R. (2015). Mechanics of intact bone marrow. J. Mech. Behav. Biomed. Mater. *50*, 299–307. https://doi.org/10.1016/j.jmbbm.2015.06.023.

39. Qin, Q., Wang, D., Xu, L., Lan, Y., and Tong, M. (2021). Evaluating Lymph Node Stiffness to Differentiate Bacterial Cervical Lymphadenitis and Lymph Node–First Presentation of Kawasaki Disease by Shear Wave Elastography. J. Ultrasound Med. *40*, 1371–1380. https://doi.org/10.1002/jum.15518.

40. Hirsch, S., Guo, J., Reiter, R., Papazoglou, S., Kroencke, T., Braun, J., and Sack, I. (2014). MR Elastography of the Liver and the Spleen Using a Piezoelectric Driver, Single-Shot Wave-Field Acquisition, and Multifrequency Dual Parameter Reconstruction. Magn. Reson. Med. *71*, 267–277. https://doi.org/10.1002/mrm.24674.

41. Dahl, K.N., Ribeiro, A.J.S., and Lammerding, J. (2008). Nuclear shape, mechanics, and mechanotransduction. Circ. Res. *102*, 1307–1318. https://doi.org/10.1161/CIRCRESAHA.108.173989.

42. Lobo, J., See, E.Y.S., Biggs, M., and Pandit, A. (2016). An insight into morphometric descriptors of cell shape that pertain to regenerative medicine. J. Tissue Eng. Regen. Med. *10*, 539–553. https://doi.org/10.1002/term.1994.

43. Maury, P., Porcel, E., Mau, A., Lux, F., Tillement, O., Mahou, P., Schanne-Klein, M.C., and Lacombe, S. (2021). Rapid Evaluation of Novel Therapeutic Strategies Using a 3D Collagen-Based Tissue-Like Model. Front. Bioeng. Biotechnol. *9*, 574035. https://doi.org/10.3389/FBIOE.2021.574035/BIBTEX.

44. Deguchi, S., Maeda, K., Ohashi, T., and Sato, M. (2005). Flow-induced hardening of endothelial nucleus as an intracellular stress-bearing organelle. J. Biomech. *38*, 1751–1759. https://doi.org/10.1016/j.jbiomech.2005.06.003.

45. Athanasiou, K.A., Eswaramoorthy, R., Hadidi, P., and Hu, J.C. (2013). Self-organization and the self-assembling process in Tissue engineering. Annu. Rev. Biomed. Eng. *15*, 115–136. https://doi.org/10.1146/annurev-bioeng-071812-152423.

46. Karamanos, N.K., Theocharis, A.D., Piperigkou, Z., Manou, D., Passi, A., Skandalis, S.S., Vynios, D.H., Orian-Rousseau, V., Ricard-Blum, S., Schmelzer, C.E.H., et al. (2021). A guide to the composition and functions of the extracellular matrix. FEBS J. *288*, 6850–6912. https://doi.org/10.1111/febs.15776.

47. Leckband, D.E., and de Rooij, J. (2014). Cadherin adhesion and mechanotransduction. Annu. Rev. Cell Dev. Biol. *30*, 291–315. https://doi.org/10.1146/annurev-cellbio-100913-013212.

48. Niessen, C.M., Leckband, D., and Yap, A.S. (2011). Tissue Organization by Cadherin Adhesion Molecules: Dynamic Molecular and Cellular Mechanisms of Morphogenetic Regulation. Physiol. Rev. *91*, 691–731. https://doi.org/10.1152/physrev.00004.2010.

49. Stolberg, S., and Mccloskey, K.E. (2009). Can Shear Stress Direct Stem Cell Fate? Biotechnol. Prog. *25*, 10–19. https://doi.org/10.1021/bp.124.

50. Jiao, Y., Zhang, Y., Xiao, Y., Xing, Y., Cai, Z., Wang, C., Zhou, Z., Feng, Z., and Gu, Y. (2022). The crescendo pulse frequency of shear stress stimulates the endothelialization of bone marrow mesenchymal stem cells on the luminal surface of decellularized scaffold in the bioreactor. Bioengineered *13*, 7925–7938. https://doi.org/10.1080/21655979.2022.2039502.

51. Slukvin, I.I., and Vodyanik, M. (2011). Endothelial origin of mesenchymal stem cells. Cell Cycle *10*, 1370–1373. https://doi.org/10.4161/cc.10.9.15345.

52. Anerillas, L.O., Kingham, P.J., Lammi, M.J., Wiberg, M., and Kelk, P. (2021). Three-dimensional osteogenic differentiation of bone marrow mesenchymal stem cells promotes matrix metallopeptidase 13 (Mmp13) expression in type i collagen hydrogels. Int. J. Mol. Sci. *22*, 13594. https://doi.org/10.3390/ijms222413594.

53. Grellier, M., Bareille, R., Bourget, C., and Amédée, J. (2009). Responsiveness of human bone marrow stromal cells to shear stress. J. Tissue Eng. Regen. Med. *3*, 302–309. https://doi.org/10.1002/TERM.166.

54. Patten, J., and Wang, K. (2021). Fibronectin in development and wound healing. Adv. Drug Deliv. Rev. *170*, 353–368. https://doi.org/10.1016/j.addr.2020.09.005.

55. Reiprich, S., Akova, E., Aszódi, A., and Schönitzer, V. (2021). Hyaluronan Synthases' Expression and Activity Are Induced by Fluid Shear Stress in Bone Marrow-Derived Mesenchymal Stem Cells. Int. J. Mol. Sci. *22*, 1–18. https://doi.org/10.3390/IJMS22063123.

56. Gu, Y., Sorokin, L., Durbeej, M., Hjalt, T., Jönsson, J.I., and Ekblom, M. (1999). Characterization of Bone Marrow Laminins and Identification of 5-Containing Laminins as Adhesive Proteins for Multipotent Hematopoietic FDCP-Mix Cells. Blood *93*, 2533–2542.

57. Textor, J., Mandl, J.N., and de Boer, R.J. (2016). The Reticular Cell Network: A Robust Backbone for Immune Responses. PLoS Biol. *14*, e2000827. https://doi.org/10.1371/journal.pbio.2000827.

58. Fletcher, A.L., Acton, S.E., and Knoblich, K. (2015). Lymph node fibroblastic reticular cells in health and disease. Nat. Rev. Immunol. *15*, 350–361. https://doi.org/10.1038/nri3846.

59. De Smet, F., Segura, I., De Bock, K., Hohensinner, P.J., and Carmeliet, P. (2009). Mechanisms of vessel branching: Filopodia on endothelial tip cells lead the way. Arterioscler. Thromb. Vasc. Biol. *29*, 639–649. https://doi.org/10.1161/ATVBAHA.109.185165.

60. Tzima, E., Irani-Tehrani, M., Kiosses, W.B., Dejana, E., Schultz, D.A., Engelhardt, B., Cao, G., DeLisser, H., and Schwartz, M.A. (2005). A mechanosensory complex that mediates the endothelial cell response to fluid shear stress. Nature *437*, 426–431. https://doi.org/10.1038/nature03952.

61. Roux, E., Bougaran, P., Dufourcq, P., and Couffinhal, T. (2020). Fluid Shear Stress Sensing by the Endothelial Layer. Front. Physiol. *11*, 861. https://doi.org/10.3389/fphys.2020.00861.

62. Yamashiro, S., Yamakita, Y., Ono, S., and Matsumura, F. (1998). Fascin, an Actin-bundling Protein, Induces Membrane Protrusions and Increases Cell Motility of Epithelial Cells. Mol. Biol. Cell *9*, 993–1006.

63. Polacheck, W.J., German, A.E., Mammoto, A., Ingber, D.E., and Kamm, R. D. (2014). Mechanotransduction of fluid stresses governs 3D cell migration. Proc. Natl. Acad. Sci. USA *111*, 2447–2452. https://doi.org/10.1073/pnas.1316848111.

64. Hubbard, A.K., and Rothlein, R. (2000). Forum: Signal Transduction Intercellular Adhesion Molecule-1 (ICAM-1) Expression And Cell Signaling Cascades. Free Radic. Biol. Med. *28*, 1379–1386.

65. Marchese, M.E., Berdnikovs, S., and Cook-Mills, J.M. (2012). Distinct sites within the vascular cell adhesion molecule-1 (VCAM-1) cytoplasmic domain regulate VCAM-1 activation of calcium fluxes versus Rac1 during leukocyte transendothelial migration. Biochemistry *51*, 8235–8246. https://doi.org/10.1021/bi300925r.

66. Moazzam, F., Delano, F.A., Zweifach, B.W., and Schmid-Schönbein, G. W. (1997). The leukocyte response to fluid stress (pseudopodadhesion-ion channelsdeformabilityshear stress). Medical Sciences *94*, 5338–5343.

67. Stacchini, A., Aragno, M., Vallario, A., Alfarano, A., Circosta, P., Gottardi, D., Faldella, A., Rege-Cambrin, G., Thunberg, U., Nilsson, K., and Caligaris-Cappio, F. (1999). MEC1 and MEC2: two new cell lines derived from B-chronic lymphocytic leukaemia in prolymphocytoid transformation. Leuk. Res. *23*, 127–136.

68. ten Hacken, E., Sivina, M., Kim, E., O'Brien, S., Wierda, W.G., Ferrajoli, A., Estrov, Z., Keating, M.J., Oellerich, T., Scielzo, C., et al. (2016). Functional Differences between IgM and IgD Signaling in Chronic Lymphocytic Leukemia. J. Immunol. *197*, 2522–2531. https://doi.org/10.4049/jimmunol.1600915.

69. Sarfati, M. (1993). CD23 and chronic lymphocytic leukemia. Blood Cells (NY) *19*, 591–599. https://pubmed.ncbi.nlm.nih.gov/8018941/.

70. Burgess, M., Gill, D., Singhania, R., Cheung, C., Chambers, L., Renyolds, B.A., Smith, L., Mollee, P., Saunders, N., and McMillan, N.A. (2013). CD62L as a therapeutic target in chronic lymphocytic leukemia. Clin. Cancer Res. *19*, 5675–5685. https://doi.org/10.1158/1078-0432.CCR-13-1037.

71. Montraveta, A., Lee-Vergés, E., Roldán, J., Jiménez, L., Cabezas, S., Clot, G., Pinyol, M., Xargay-Torrent, S., Rosich, L., Arimany-Nardí, C., et al. (2015). CD69 expression potentially predicts response to bendamustine and its modulation by ibrutinib or idelalisib enhances cytotoxic effect in chronic lymphocytic leukemia. Oncotarget *7*, 5507–5520. https://doi.org/10.18632/oncotarget.6685.

72. Tissino, E., Benedetti, D., Herman, S.E.M., ten Hacken, E., Ahn, I.E., Chaffee, K.G., Rossi, F.M., Dal Bo, M., Bulian, P., Bomben, R., et al. (2018). Functional and clinical relevance of VLA-4 (CD49d/CD29) in ibrutinib-treated chronic lymphocytic leukemia. J. Exp. Med. *215*, 681–697. https://doi.org/10.1084/jem.20171288.

73. Zhang, S., Wu, C.C.N., Fecteau, J.F., Cui, B., Chen, L., Zhang, L., Wu, R., Rassenti, L., Lao, F., Weigand, S., and Kipps, T.J. (2013). Targeting chronic lymphocytic leukemia cells with a humanized monoclonal antibody specific for CD44. Proc. Natl. Acad. Sci. USA *110*, 6127–6132. https://doi.org/10.1073/pnas.1221841110.

74. Ibrahim, S., Keating, M., Do, K.-A., O'brien, S., Huh, Y.O., Jilani, I., Lerner, S., Kantarjian, H.M., and Albitar, M. (2001). CD38 expression as an important prognostic factor in B-cell chronic lymphocytic leukemia. Blood *98*, 181–186. https://doi.org/10.1182/blood.v98.1.181.

75. Scielzo, C., Apollonio, B., Scarfò, L., Janus, A., Muzio, M., Ten Hacken, E., Ghia, P., and Caligaris-Cappio, F. (2011). The functional in vitro response to CD40 ligation reflects a different clinical outcome in patients with chronic lymphocytic leukemia. Leukemia *25*, 1760–1767. https://doi.org/10.1038/leu.2011.149.

76. Burger, J.A., Burger, M., and Kipps, T.J. (1999). Chronic Lymphocytic Leukemia B Cells Express Functional CXCR4 Chemokine Receptors That Mediate Spontaneous Migration Beneath Bone Marrow Stromal Cells. Blood *94*, 3658–3667. https://doi.org/10.1182/blood.V94.11.3658.

77. Cuesta-Mateos, C., Brown, J.R., Terrón, F., and Muñoz-Calleja, C. (2021). Of Lymph Nodes and CLL Cells: Deciphering the Role of CCR7 in the Pathogenesis of CLL and Understanding Its Potential as Therapeutic Target. Front. Immunol. *12*, 662866. https://doi.org/10.3389/fimmu.2021.662866.

78. Zhang, J., Wehrle, E., Adamek, P., Paul, G.R., Qin, X.H., Rubert, M., and Müller, R. (2020). Optimization of mechanical stiffness and cell density of 3D bioprinted cell-laden scaffolds improves extracellular matrix mineralization and cellular organization for bone tissue engineering. Acta Biomater. *114*, 307–322. https://doi.org/10.1016/j.actbio.2020.07.016.

CellPress
OPEN ACCESS

79. Pittenger, M.F., Discher, D.E., Péault, B.M., Phinney, D.G., Hare, J.M., and Caplan, A.I. (2019). Mesenchymal stem cell perspective: cell biology to clinical progress. NPJ Regen. Med. *4*, 22. https://doi.org/10.1038/s41536-019-0083-6.

80. Bankhead, P., Loughrey, M.B., Fernández, J.A., Dombrowski, Y., McArt, D.G., Dunne, P.D., McQuaid, S., Gray, R.T., Murray, L.J., Coleman, H. G., et al. (2017). QuPath: Open source software for digital pathology image analysis. Sci. Rep. *7*, 16878. https://doi.org/10.1038/s41598-017-17204-5.

81. Schindelin, J., Arganda-Carreras, I., Frise, E., Kaynig, V., Longair, M., Pietzsch, T., Preibisch, S., Rueden, C., Saalfeld, S., Schmid, B., et al. (2012). Fiji: An open-source platform for biological-image analysis. Nat. Methods *9*, 676–682. https://doi.org/10.1038/nmeth.2019.

STAR★METHODS

KEY RESOURCES TABLE

REAGENT or RESOURCE	SOURCE	IDENTIFIER
Antibodies		
Rabbit Polyclonal CD31 antibody	Abcam	CAT#ab32457; RRID: AB_726369
Knockout Tested Rabbit Recombinant Monoclonal CD90/Thy1 antibody	Abcam	CAT# b307736
Rabbit Polyclonal ALP antibody	Thermo Fisher Scientific	CAT#PA5-56239 RRID: AB_2637878
Collagen IV Monoclonal Antibody (1042), Alexa Fluor™ 488, eBioscience™	Thermo Fisher Scientific	CAT#53-9871-82 RRID: AB_2574487
Rabbit Recombinant Monoclonal COL4A1 antibody	Abcam	CAT#ab236640
Fibronectin Monoclonal Antibody (FN-3), Alexa Fluor™ 488, eBioscience™	Thermo Fisher Scientific	CAT#53-9869-82 RRID: AB_11063704
Mouse monoclonal Laminin alpha4 antibody	BioTechne	CAT# MAB7340
Rabbit Polyclonal Laminin alpha 5 antibody	Thermo Fisher Scientific	CAT# BS-1086R
Rabbit Polyclonal Pan-Cadherin antibody	Abcam	CAT# ab16505 RRID:AB_443397
Mouse Monoclonal Podoplanin antibody	Abcam	CAT# ab10288 RRID:AB_297027
VE-Cadherin (D87F2) XP® Rabbit mAb	CellSignalling	CAT#2500S
Donkey anti-Rabbit IgG (H + L) Highly Cross-Adsorbed Secondary Antibody, Alexa Fluor™ Plus 488	Thermo Fisher Scientific	Cat# A32790 RRID:AB_2762833
Donkey anti-Mouse IgG (H + L) Highly Cross-Adsorbed Secondary Antibody, Alexa Fluor™ Plus 488	Thermo Fisher Scientific	CAT#A32766 RRID:AB_2762823
Goat anti-Rabbit IgG (H + L) Highly Cross-Adsorbed Secondary Antibody, Alexa Fluor™ Plus 647	Thermo Fisher Scientific	CAT# A32733 RRID:AB_2633282
Goat anti-Mouse IgG (H + L) Highly Cross-Adsorbed Secondary Antibody, Alexa Fluor™ Plus 647	Thermo Fisher Scientific	CAT# A32728 RRID:AB_2633277
Donkey anti-Rabbit IgG (H + L) Highly Cross-Adsorbed Secondary Antibody, Alexa Fluor™ 568	Thermo Fisher Scientific	CAT# A10042 RRID:AB_2534017
Goat anti-Mouse IgG (H + L) Highly Cross-Adsorbed Secondary Antibody, Alexa Fluor™ 568	Thermo Fisher Scientific	CAT# A11031 RRID:AB_144696
IgD Antibody, anti-human, REAfinity™	Miltenyi	CAT#130-110-648 RRID:AB_2652266
IgM Antibody, anti-human	Miltenyi	CAT#130-122-915 RRID:AB_2801965
CD184 (CXCR4) Antibody, anti-human, REAfinity™	Miltenyi	CAT#130-120-708 RRID:AB_2752173
CD197 (CCR7) Antibody, anti-human, REAfinity™	Miltenyi	CAT#130-117-353 RRID:AB_2733933
CD19 Antibody, anti-human, REAfinity™	Miltenyi	CAT#130-114-522 RRID:AB_2751237
CD19 Antibody, anti-human, REAfinity™	Miltenyi	CAT#130-113-647 RRID:AB_2726200
BD Pharmingen™ PE Mouse Anti-Human CD23	BD Bioscience	CAT#555711 RRID:AB_396056
CD29 Antibody, anti-human, REAfinity™	Miltenyi	CAT#130-118-122 RRID:AB_2751454
CD31 Antibody, anti-human, REAfinity™	Miltenyi	CAT#130-110-669 RRID:AB_2657281

(*Continued on next page*)

CellPress
OPEN ACCESS

Continued

REAGENT or RESOURCE	SOURCE	IDENTIFIER
BD Pharmingen™ PE Mouse Anti-Human CD38	BD Bioscience	CAT#555460 RRID:AB_395853
CD40 Antibody, anti-human	Miltenyi	CAT#130-123-798 RRID:AB_2811556
CD44 Antibody, anti-human, REAfinity™	Miltenyi	CAT#130-113-338 RRID:AB_2726114
CD45 Antibody, anti-human, REAfinity™	Miltenyi	CAT#130-110-637 RRID:AB_2658243
CD49d Antibody, anti-human, REAfinity™	Miltenyi	CAT#130-127-192 RRID:AB_2905060
CD62L Antibody, anti-human	Miltenyi	CAT#130-113-617 RRID:AB_2733392
CD69 Antibody, anti-human, REAfinity™	Miltenyi	CAT#130-112-610 RRID:AB_2659069
CD73 Antibody, anti-human, REAfinity™	Miltenyi	CAT#130-111-909 RRID:AB_2659167
CD80 Antibody, anti-human, REAfinity™	Miltenyi	CAT#130-128-249 RRID:AB_2921838
**Bacterial and virus strains**		
rLV-EF1α-AmCyan1-IRES-Puro-WPRE vector	Vectalis	CAT#0011VCT
rLV-EF1α-mCherry-IRES-Puro-WPRE vector	Vectalis	CAT#0009VCT
**Chemicals, peptides, and recombinant proteins**		
Hyaluronic Acid Binding Protein, Bovine Nasal Cartilage, Biotinylated	Sigma Aldrich	CAT#385911
Streptavidin, Alexa Fluor™ 647 conjugate	Thermo Fisher Scientific	CAT# S32357
Phalloidin–Atto 565	Sigma Aldrich	CAT#94072
Hoechst 33342	Merck	CAT#23491-52-3
Emallume Mayer	Bio Optica	CAT#05-06002
Eosina G soluzione acquosa 1%	Bio Optica	CAT#05-10007
TaqMan™ Fast Advanced Master Mix	Applied Biosystems	CAT#4304437
**Critical commercial assays**		
Alamar Blue® assay	Thermo Fisher Scientific	CAT# DAL1025
LIVE/DEAD Cell Imaging Kit (488/570)	Thermo Fisher Scientific	CAT# R37601
ReliaPrep™ RNA Tissue Miniprep System	Promega	CAT# Z6111
ReliaPrep™ RNA Cell Miniprep System	Promega	CAT# Z6011
RevertAid® H Minus First Strand cDNA Synthesis Kit	Thermo Fisher Scientific	CAT# K1622
Liberase TL	Merck	CAT#05401020001
Hyaluronidase	Sigma Aldrich	CAT# H4272
LIVE/DEAD™ Fixable Green Dead Cell Stain Kit, for 488 nm excitation	Thermo Fisher Scientific	CAT# L34969
**Deposited data**		
Raw and analyzed data	This paper	Available from the corresponding author on request
**Experimental models: cell lines**		
MEC-1	DSMZ	CAT#ACC497 RRID:CVCL_1870
hMSC-TERT292	Evercyte	CAT# CHT-063-0292
Human umbilical vein endothelial cells (HUVECs)	Lonza	CAT#00191027
Human lymphatic fibroblast (HLF)	ScienCell	CAT#2530
**Oligonucleotides**		
FSCN1 (Hs00602051_mH)	Thermo Fisher Scientific	CAT# 4331182
LMNA (Hs00153462_m1)	Thermo Fisher Scientific	CAT# 4331182
PTK2 (Hs01056457_m1)	Thermo Fisher Scientific	CAT#4331182
PECAM1 (CD31) (Hs01065290_m1)	Thermo Fisher Scientific	CAT# 4351372
ICAM-1 (Hs00164932_m1)	Thermo Fisher Scientific	CAT#4331182

(*Continued on next page*)

Continued

REAGENT or RESOURCE	SOURCE	IDENTIFIER
VCAM-1 (Hs00365485_m1)	Thermo Fisher Scientific	CAT#4331182
GAPDH (Hs03929097_g1)	Thermo Fisher Scientific	CAT# 4331182
Software and algorithms		
SimFlow	SimFlow Technologies	https://sim-flow.com/download/cfd-simulation-software/
SolidWorks	Dassault Systèmes	https://www.solidworks.com/
Fiji	Schindelin, J et al.[1]	https://fiji.sc/
Arivis	Zeiss	https://www.zeiss.com/microscopy/en/products/software/advanced-image-analysis.html
Huygens	Scientific Volume Imaging	https://svi.nl/Huygens-Software
FCS express 7	*De Novo* Software	https://denovosoftware.com/
QuPath	Bankhead, P. et al.[2]	https://qupath.github.io/
CFX Maestro Software	BioRad	https://www.bio-rad.com/it-it/product/cfx-maestro-software-for-cfx-real-time-pcr-instruments?ID=OKZP7E15
GraphPad	Dotmatics	https://www.graphpad.com/
Other		
Spongostan Dental	Ferrosan Medical Devices	CAT# MS0005
LiveFlow – peristaltic pump	IVTech *in vitro* technologies	https://www.ivtech.it/it/prodotti/pompe-peristaltiche/
LiveBox1 - bioreactor	IVTech *in vitro* technologies	https://www.ivtech.it/it/prodotti/bioreattori/
LiveBox2 - bioreactor	IVTech *in vitro* technologies	https://www.ivtech.it/it/prodotti/bioreattori/

EXPERIMENTAL MODEL AND STUDY PARTICIPANT DETAILS

HLF cell culture

Human lymphatic fibroblast (HLF) primary cells were obtained from ScienCell (Cat. No. 2530; ScienCell, Carlsbad, California). Cells were cultured at 37°C with 5% CO_2 with and used at a passage number below 10. Fibroblast medium (FM) was supplemented with 2% fetal bovine serum (FBS), 1% fibroblast growth supplement (FGS), and 1% antibiotic solution (ScienCell, Carlsbad, California). Culture flasks were pre-coated with poly-L-lysine (Cat. No. P4707; Sigma-Aldrich, Missouri, USA) at a dilution of 15μL in 10mL of sterile water and incubated for 1 h at 37°C. Donor information for HLF cells was not specified by ScienCell.

HUVEC cell culture

Human umbilical vein endothelial cells (HUVECs) were obtained from Lonza (Cat. No. 00191027; Lonza, Basel, Switzerland). Cells were cultured at 37°C with 5% CO_2 and used at a passage number below 10. The complete medium EGM-2 BulletKit (Cat. No. CC-3162; Lonza, Basel, Switzerland) was used. Culture flasks were pre-coated with 1.8% gelatin from bovine skin (Cat. No. G9391; Sigma-Aldrich, Missouri, USA) in sterile water. HUVECs were pooled from different donors.

hMSC cell culture

Immortalized mesenchymal stem cells (hMSC-TERT292) were obtained from Evercyte (Cat. No. CHT-063-0292; Evercyte GmbH, Vienna, Austria). Cells were cultured at 37°C with 5% CO_2 in complete medium using the MesenCult-ACF Plus Culture Kit (Cat. No. 05445; StemCell Technologies, Vancouver, Canada). Culture flasks were pre-coated with the coating reagent provided in the kit (Cat. No. G9391; Sigma-Aldrich, Missouri, USA) and incubated for 2 h at room temperature.

MEC1 cell culture

The CLL cell line MEC1 was obtained from DSMZ (Cat. No. ACC 497; DSMZ, Braunschweig, Germany). Cells were cultured at 37°C with 5% CO_2 in RPMI medium (Cat. No. ECB2000; Euroclone, Milano, Italy) supplemented with 10% fetal bovine serum (Cat. No. FBS-11A; CliniSciences, Amsterdam, Netherlands) and 0.04% gentamicin (Cat. No. G1397; Sigma-Aldrich, Missouri, USA).

METHOD DETAILS

Biomaterial characterization: Spongostan

Spongostan was obtained in sterile single cubes of 1 × 1 × 1cm and then cut in cylinders of 5 mm in height and 8 mm in diameter (Cat. No. MS0005 - Ferrosan Medical Devices A/S, Søborg, Denmark).

Porosity

Porosity was measured using the liquid displacement method, based on the principle that a submerged object displaces a volume of liquid equal to its own volume. A defined initial volume (V_1) of liquid was placed in a beaker, followed by insertion of the dry scaffold to obtain the total volume (V_2). After scaffold removal, the remaining liquid volume (V_3) was measured. The number of scaffolds used was $n = 3$ (Figure 1B).

$$p(\%) = \frac{(V1 - V3)}{(V2 - V3)} x100$$

Topography and pore dimensions

The internal topography of the Spongostan scaffold is heterogeneous, with a wide range of pore sizes. The structure of unstained collagen scaffolds was qualitatively visualized by second harmonic generation (SHG) imaging using a 4TuneDual-IR DIVE STELLARIS 8 FALCON multiphoton microscope (Leica Microsystems GmbH, Wetzlar, Germany) at 850 nm two-photon excitation. Emission in the range of 420–430 nm was collected by a spectral 4Tune single-photon counting NDD detector. SHG images were acquired from dry samples using a 10×0.40 HC PL APO CS dry objective.

For quantitative assessment of average pore size, scaffolds were soaked in PBS, embedded in Killik O.C.T. cryo-embedding compound (Cat. No. 05–9801; Bio-Optica, Milan, Italy), and sectioned into 30 μm slices using a cryostat. z stack reconstructions of the slices were acquired using a TCS SP8 confocal microscope (Leica Microsystems GmbH, Wetzlar, Germany). Pore segmentation and 3D reconstruction were performed using the Arivis Scientific Image Analysis Platform (Zeiss, Oberkochen, Germany) to measure pore size frequency distribution and weighted average diameter ($n = 3$ scaffolds) (Figures 1C and 1D).

Compressive modulus

The compressive mechanical properties of dry and hydrated Spongostan samples ($n = 3$ per type) were tested using a Dynamic Mechanical Analyzer (DMA Q800; TA Instruments). All tests were performed at room temperature with a preload of 0.001 N. Each analysis included a loading phase at 2.5% min-1 up to 30% compression strain, followed by an unloading phase at 5% min-1 until 1% strain. Stress–strain curves were used to calculate the compressive modulus (Young's modulus) as the slope of the loading curve in the linear region (Figure 1E).

Cell transduction

To generate fluorescent HLF-cyan and HUVEC-red cells, Vectalis constitutive lentiviral vectors and the associated transduction protocol were used. The rLV-EF1α-AmCyan1-IRES-Puro-WPRE vector (Cat. No. 0011VCT; Vectalis, Toulouse, France) was used for HLFs, and the rLV-EF1α-mCherry-IRES-Puro-WPRE vector (Cat. No. 0009VCT; Vectalis, Toulouse, France) was used for HUVECs. Figure 1H.

3D scaffold generation

To generate the 3D culture, the dry scaffold (cylinder 5 × 8 mm, V = 0.25 cm^3) was placed in a 1.5 mL centrifuge tube, positioned just above the tube's midpoint to leave space beneath. Cells were resuspended in 200 μL of medium and the suspension was carefully applied as a drop onto the top of the scaffold inside the tube. The scaffold was incubated for 2–3 h at 37°C with 5% CO_2 to allow complete absorption of the medium and enable cell infiltration and adhesion to the matrix. After this period, 800 μL of medium were added to the tube, and the 3D cultures were maintained at 37°C with 5% CO_2. Static scaffolds remained in the centrifuge tubes, while scaffolds designated for dynamic conditions were transferred into bioreactors the day after seeding. A total of 6×10^5 cells were seeded per scaffold for both BM and LN models. For the LN co-culture, HLF and HUVEC cells were seeded at a 1:2 ratio (2×10^5 HLF and 4×10^5 HUVEC) and maintained in a 1:1 mixture of FM and EGM-2 medium (Figure 1F).

Live/dead assay

To assess cell viability, static scaffolds were stained using the LIVE/DEAD Cell Imaging Kit (488/570) (Cat. No. R37601; Thermo Fisher Scientific, Massachusetts, USA) and evaluated at 1, 5, and 15 days post-seeding. Scaffolds were washed with DMEM without serum and phenol red (Cat. No. 21063029; Thermo Fisher Scientific, Massachusetts, USA). The LIVE/DEAD stock solution was diluted 1:3 in the same medium and applied to the scaffolds. Live and dead cells were visualized, and z stack reconstructions were (Figure 1G).

Alamar Blue assay

The Alamar Blue assay (Cat. No. DAL1025; Thermo Fisher Scientific, Massachusetts, USA) was performed on static 3D scaffolds after 1, 5, and 15 days of culture. The reagent was diluted 1:10 in the appropriate culture medium (see 3D scaffold generation), added to the scaffolds, and incubated at 37°C with 5% CO_2 for 4 h. As a control for background fluorescence, an empty scaffold was incubated with both medium and Alamar Blue solution. After incubation, 100 μL of the mixture was transferred to a 96-well light-screened

plate, and relative fluorescence units (RFU) were measured using the Victor3 multilabel plate reader (PerkinElmer, Buckinghamshire, UK). Background fluorescence was subtracted from the absolute RFU values (Figure S1).

Dynamic system

Scaffolds maintained under dynamic conditions were transferred into the system the day after seeding. The dynamic apparatus included the LiveFlow system (IVTech *in vitro* technologies, Lucca, Italy), a peristaltic pump with two independent heads capable of applying different flow rates simultaneously. The circuit consisted of a reservoir containing 10 mL of the specific culture medium and a bioreactor. For the LN model, the LB2 bioreactor (IVTech *in vitro* technologies, Lucca, Italy) was used (wet volume: 2.5 mL). This bioreactor comprises two chambers separated by a hydrophilic nylon membrane with a pore size of 100μm (Merck KGaA, Darmstadt, Germany). The scaffold was placed in the upper chamber and exposed to a bottom-to-top sigmoidal flow at a rate of 200 μL/min. The BM model was cultured in the LB1 bioreactor (IVTech *in vitro* technologies, Lucca, Italy), which consists of a single chamber (wet volume: 1.5 mL). The scaffold was placed within the chamber and stimulated with a tangential flow at a rate of 100 μL/min (Figure 2A).

CLL cell line recirculation in the dynamic system

MEC1 cells were resuspended in the circulating medium (RPMI) at a concentration of 5×10^5/mL. The dynamic configuration used for this step, referred to as the "niche" conformation, included the LB2 bioreactor (for both LN and BM models), with the scaffold placed in the upper chamber. A tangential flow was applied only in the upper chamber at a rate of 100 μL/min (Figure 2C).

Computational fluid dynamic study (CFD)

The CFD study was performed by the IVTech *in vitro* technologies team. Fluid dynamics within the chambers was simulated using SimFlow software (SimFlow Technologies), and scaffold geometry was designed with SolidWorks (Dassault Systèmes).

The scaffold used in the system was consistently a cylinder measuring 5 mm in height and 8 mm in diameter. This cylindrical shape (Figure 1A) was advantageous in minimizing edge effects caused by medium flow impacting the lateral surface. In the CFD simulations, the matrix was considered non-porous, allowing for evaluation of the effects of the dynamic environment on the external scaffold walls. For the LB1 configuration, the scaffold was modeled as lying at the bottom of the chamber, and shear stress (SS) acting on the top and lateral walls was analyzed at a flow rate of 100 μL/min. In the LB2 configuration, the scaffold was positioned in the upper chamber, lying on the membrane, and SS was evaluated on the bottom and lateral walls at a flow rate of 200 μL/min (Figure 2B). In the "niche" conformation, the scaffold was similarly placed in the upper chamber on the membrane, and SS was analyzed on the top, bottom, and lateral walls at a flow rate of 100 μL/min. The geometry was discretized into elementary cells to create a mesh model, enabling precise representation of the local dynamics. This method provides greater accuracy than estimating average SS across the whole scaffold. The fluidic model used viscosity and density parameters corresponding to water at 37°C, and the liquid was assumed to flow continuously at various flow rates (50, 100, 200, and 300 μL/min) (Figure 3). The flow was characterized by streamlines oriented normally to the internal cross-section of the tubing.

Scaffold processing for imaging analysis

At the end of maturation (15 days post-seeding) or following CLL cell recirculation (15 days of maturation plus 72 h of circulation), scaffolds were fixed in 4% paraformaldehyde solution in PBS (Cat. No. 30525-89-4; Santa Cruz, Texas, USA) for 2–3 h. Samples were then soaked in 30% sucrose solution in PBS for 45 min (Cat. No. 57-50-1; Merck KGaA, Darmstadt, Germany). Following fixation, scaffolds were embedded in Killik O.C.T. cryo embedding compound (Cat. No. 05–9801; Bio-Optica, Milan, Italy), rapidly frozen in isopentane and dry ice, and stored at −80°C. Cryosections were cut using a cryostat at 7 μm thickness for histochemistry and 30 μm thickness for confocal reconstructions.

Histochemistry

For hematoxylin and eosin staining, tissue sections were rehydrated with distilled water, stained with hematoxylin (Cat. No. 05–06002; BioOptica S.p.A., Milan, Italy), rinsed under running water, and soaked in 95% ethanol. Sections were then stained with eosin (Cat. No. 05–10007; BioOptica S.p.A., Milan, Italy), washed, and dehydrated through a graded ethanol series (95%, 100%) followed by xylene. Histochemistry images were acquired using Aperio AT2 Scanner (Leica, Wetzlar, Germany). Images were acquired using 20x/0.75NA Plan Apo (40x scanning with 2x automatic optical mag changer) objective and have a resolution of 1010× 741 pixel. Images were analyzed with QuPath.[80]

Immunofluorescence analyses

For immunofluorescence staining, slices were rehydrated with PBS, permeabilized, and blocked using a solution containing 10% FBS, 0.3% Triton X-100 (Cat. No. A4975; AppliChem GmbH, Darmstadt, Germany), and 1 mg/mL BSA (Cat. No. A2153; Sigma-Aldrich, Missouri, USA). Sections were incubated with primary antibodies overnight at 4°C, followed by secondary antibody incubation for 2 h at room temperature. Nuclei were stained with Hoechst 33342 (Cat. No. 23491-52-3; Merck KGaA, Darmstadt, Germany) at a 1:2000 dilution for 10 min at room temperature. The following primary antibodies were used: anti-CD31 (Cat. No. ab32457; Abcam, Cambridge, UK, 1:100), anti-CD90 (Cat. No. ab307736; Abcam, Cambridge, UK, 1:250), anti-ALP (Cat. No. PA5-56239; Thermo Fisher Scientific, Massachusetts, USA, 1:50), anti-collagen IV (Cat. No. 53-9871-82; Thermo Fisher Scientific, Massachusetts, USA,

1:50), anti-collagen IV (Cat. No. ab236640; Abcam, Cambridge, UK, 1:100), anti-fibronectin (Cat. No. 53-9869-82; Thermo Fisher Scientific, Massachusetts, USA, 1:100), hyaluronic acid binding protein biotinylated (Cat. No. 385911; Sigma-Aldrich, Missouri, USA,1:200), anti-laminin 4α (Cat. No. MAB7340; BioTechne, Minneapolis, US, 1:200), anti-laminin 5α (Cat. No. BS-1086R; Thermo Fisher Scientific, Massachusetts, USA, 1:100), anti-pan-cadherin (Cat. No. ab16505, Abcam; Cambridge, UK, 1:250), anti-podoplanin (Cat. No. ab10288; Abcam, Cambridge, UK, 1:100), anti-ve-cadherin (Cat. No. 2500S; CellSignalling, Massachusetts, US, 1:200). Secondary antibodies used: Alexa Fluor 488 (Cat. No. A32790 and A32766; Thermo Fisher Scientific, Massachusetts, USA, 1:500), Alexa Fluor 647 (Cat. No. A32733 and A32728; Thermo Fisher Scientific, Massachusetts, USA, 1:500), Alexa Fluor 568 (Cat. No. A10042 and A11031; Thermo Fisher Scientific, Massachusetts, USA, 1:500), Streptavidin Alexa Fluor 647 Conjugate (Cat. No. S32357; Thermo Fisher Scientific, Massachusetts, USA, 1:500). Actin was stained using Alexa Fluor 568 Phalloidin, added with secondary antibodies (Cat. No. 94072; Sigma-Aldrich, Missouri, USA, 1:250). Immunofluorescence images from Figures 1G, 3, 4, 5, and 7 and Video S2 were acquired as z stack reconstructions using Olympus FluoVIEW 3000 RS confocal microscope (Olympus, Tokyo, Japan). Emission of 405, 488, 561, 640 nm was collected by PMT detectors. All images have a resolution of 1024 x 1024 pixels. Images were acquired using 10X (NA 0.40; voxel size:1,243 x 1,243 x 1.5 μm) Dry, 20X (NA 0.8; voxel size: 0.621 x 0.621 x 0.5 μm) Dry, 30X (NA 1.05; voxel size: 0.414 x 0.414 x 0.5 μm) Sil and 60X (NA 1.3; voxel size: 0.207 x 0.207 x 0.5μm) Sil objectives. Figure 7A(b) has been acquired with a 60x objective with a 1.5 zoom (voxel size: 0.138 x 0.138 x 0.5 μm). Immunofluorescence images from Figures 1C and 1H were acquired as z stack reconstructions using Leica TCS SP8 SMD FLIM Laser Scanning Confocal (Leica, Wetzlar, Germany). Emission of 405nm was collected by a PMT detector; 488 and 568 nm was collected by HyBrid detectors. All images have a resolution of 1024 x 1024 pixels. Images were acquired using 10x (NA 0.40; voxel size: 1,136 x 1,136 × 1 μm) Dry, 20X (NA 0.75; voxel size: 0.757 x 0.757 x 1.5 μm) Dry and 40X (NA 1.3; voxel size: 0.284 x 0.284 x 1.5 μm) Oil objectives. The staining with the same markers was repeated on slices taken from different scaffold depths to assess the distribution of specific markers. Images were analyzed with Fiji[81] and Arivis Scientific Image Analysis Platform (Zeiss, Oberkochen, Germany) softwares. Alle the image analysis have been made on z stack reconstructions and images shown in the figures are maximum projections.

Morphological confocal analysis

Morphological analysis of cells grown within the scaffolds was performed using Huygens software (Scientific Volume Imaging, Hilversum, Netherlands). Images used for the analysis were obtained from sections stained with Hoechst 33342 (Cat. No. 23491-52-3; Merck KGaA, Darmstadt, Germany; 1:2000). Cell nuclei from the reconstructed 3D images were segmented and analyzed for object sphericity and minimum distance to the nearest neighbor. Minimum distance was calculated between the centers of mass (CM) of the 3D segmented nuclear structures. For each condition (LN static, LN dynamic, BM static, BM dynamic), three biological replicates were analyzed. For each replicate, three slices at different scaffold depths were selected, and two distinct image acquisitions from separate regions of each slice were examined. Data are presented as mean ± SEM. Statistical analysis was performed using an unpaired t-test with Welch's correction ($p < 0.05$).

RNA extraction and real-time PCR

RNA extraction was performed on scaffolds after 15 days of maturation using the with ReliaPrep RNA Tissue Miniprep System (Cat. No. Z6111; Promega, Wisconsin, USA), following the manufacturer's protocol. For 2D cell cultures, RNA extraction was carried out using the ReliaPrep RNA Cell Miniprep System (Cat. No. Z6011; Promega, Wisconsin, USA). cDNA synthesis was performed using the RevertAid H Minus First Strand cDNA Synthesis Kit (Cat. No. K1622; Thermo Fisher Scientific, Massachusetts, USA), according to the manufacturer's instructions. RT-qPCR analysis was conducted using the TaqMan Fast Advanced Master Mix (Cat. No. 4304437; Applied Biosystems, Massachusetts, USA) and TaqMan gene expression probes (Applied Biosystems) on a CFX96 Real-Time PCR Detection System (Bio-Rad, California, USA). Quantification of FSCN1 (Hs00602051), LMNA (Hs00153462), PTK2 (Hs01056457), CD31 (Hs01065290), PDPN (Hs00366766), ICAM1 (Hs00164932), and VCAM1 (Hs00365485) transcripts was performed using the ΔCt method, with GAPDH (Hs03929097) as the housekeeping gene. RT-qPCR analysis was conducted on both LN and BM models under 3D static and dynamic conditions, and on 2D co-cultures of HLF and HUVEC cells (LN) or monocultures of hMSCs (BM).

Scaffold processing for flow cytometry

3D LN scaffolds were dissociated for flow cytometry analysis. Samples were placed in 1 mL of dissociation buffer prepared by adding liberase (Cat. No. 05401020001; Merck KGaA, Darmstadt, Germany) and hyaluronidase (Cat. No. H4272; Sigma-Aldrich, Missouri, USA) to PBS containing $MgCl_2$ and $CaCl_2$ (Cat. No. D8662; Sigma-Aldrich, Missouri, USA), at final concentrations of 25 μg/mL and 300 μg/mL, respectively. Static samples were incubated in the solution for 10 min at 37°C, and dynamic samples for 15 min at 37°C. Following enzymatic incubation, samples were mechanically dissociated by vigorous pipetting for several minutes. Enzymatic activity was then quenched by adding fetal bovine serum (Cat. No. FBS-11A; CliniSciences, Amsterdam, Netherlands). The resulting cell suspension was subsequently filtered.

Flow cytometric analyses

MEC1 circulating cells were collected after 24 and 72 h of recirculation within the system. Stromal cells were obtained from dissociated scaffolds that had been matured for 15 days under both static and dynamic conditions. Cells were washed with PBS and incubated for 25 min with the LIVE/DEAD Fixable Green Dead Cell Stain Kit (Cat. No. L34969; Thermo Fisher Scientific, Massachusetts,

USA) and specific antibodies. Following incubation, cells were washed twice with PBS and immediately analyzed using a CytoFLEX S flow cytometer (Beckman Coulter, California, USA). The antibodies used included: IgD (Cat. No. 130-110-648; Miltenyi, Bergisch Gladbach, Germany) IgM (Cat. No. 130-122-915; Miltenyi, Bergisch Gladbach, Germany) CXCR4 (Cat. No. 130-120-708; Miltenyi, Bergisch Gladbach, Germany) CCR7 (Cat. No. 130-117-353; Miltenyi, Bergisch Gladbach, Germany) CD19 (Cat. No. 130-114-522; Miltenyi, Bergisch Gladbach, Germany), (Cat. No. 130-113-647; Miltenyi, Bergisch Gladbach, Germany) CD23 (Cat. No. 555711; BD Biosciences, New Jersey, USA) CD29 (Cat. No. 130-118-122; Miltenyi, Bergisch Gladbach, Germany) CD31 (Cat. No. 130-110-669; Miltenyi, Bergisch Gladbach, Germany) CD38 (Cat. No. 555460; BD Biosciences, New Jersey, USA) CD40 (Cat. No. 130-123-798; Miltenyi, Bergisch Gladbach, Germany) CD44 (Cat. No. 130-113-338; Miltenyi, Bergisch Gladbach, Germany) CD45 (Cat. No. 130-110-637; Miltenyi, Bergisch Gladbach, Germany) CD49d (Cat. No. 130-127-192; Miltenyi, Bergisch Gladbach, Germany) CD62L (Cat. No. 130-113-617; Miltenyi, Bergisch Gladbach, Germany) CD69 (Cat. No. 130-112-610; Miltenyi, Bergisch Gladbach, Germany) CD73 (Cat. No. 130-111-909; Miltenyi, Bergisch Gladbach, Germany) CD80 (Cat. No. 130-128-249; Miltenyi, Bergisch Gladbach, Germany). Flow cytometry data were analyzed with FCS express 7 (*De Novo* Software, Pasadena, USA)

QUANTIFICATION AND STATISTICAL ANALYSIS

GraphPad Software was used to generate all figure graphs and perform statistical analyses. Details of individual statistical tests are provided in the legends of the corresponding figures.

Cell Reports Methods, Volume *5*

Supplemental information

Dynamic stimulation promotes functional tissue-like organization of a 3D human lymphoid microenvironment model *in vitro*

Dafne Barozzi, Fiorella Scagnoli, Federica Barbaglio, Daniela Belloni, Davide Ribezzi, Silvia Farè, Valeria Berno, Riccardo Pinos, Marta Sampietro, Margherita Pauri, Barbara Vergani, Francesco Mantegazza, Paolo Ghia, and Cristina Scielzo

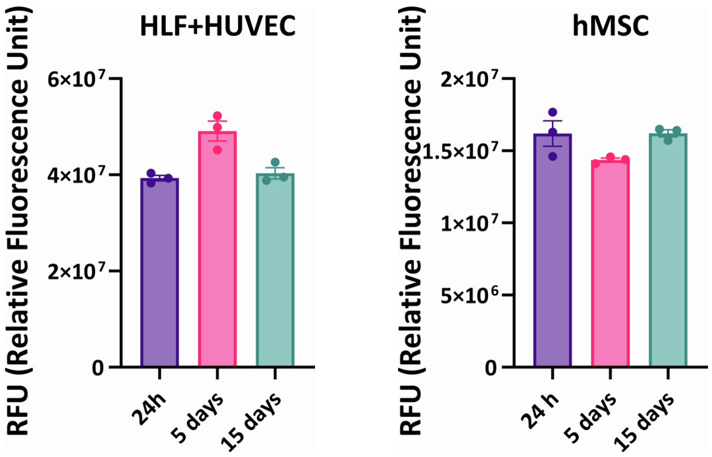

Supplementary Figure 1 – AlamarBlue viability assay, **related to Figure 1** AlamarBlue cell viability assay fluorescence intensity values showing metabolic activity of the 3D cultures over time (n=3). All data are represented by mean ± SEM.

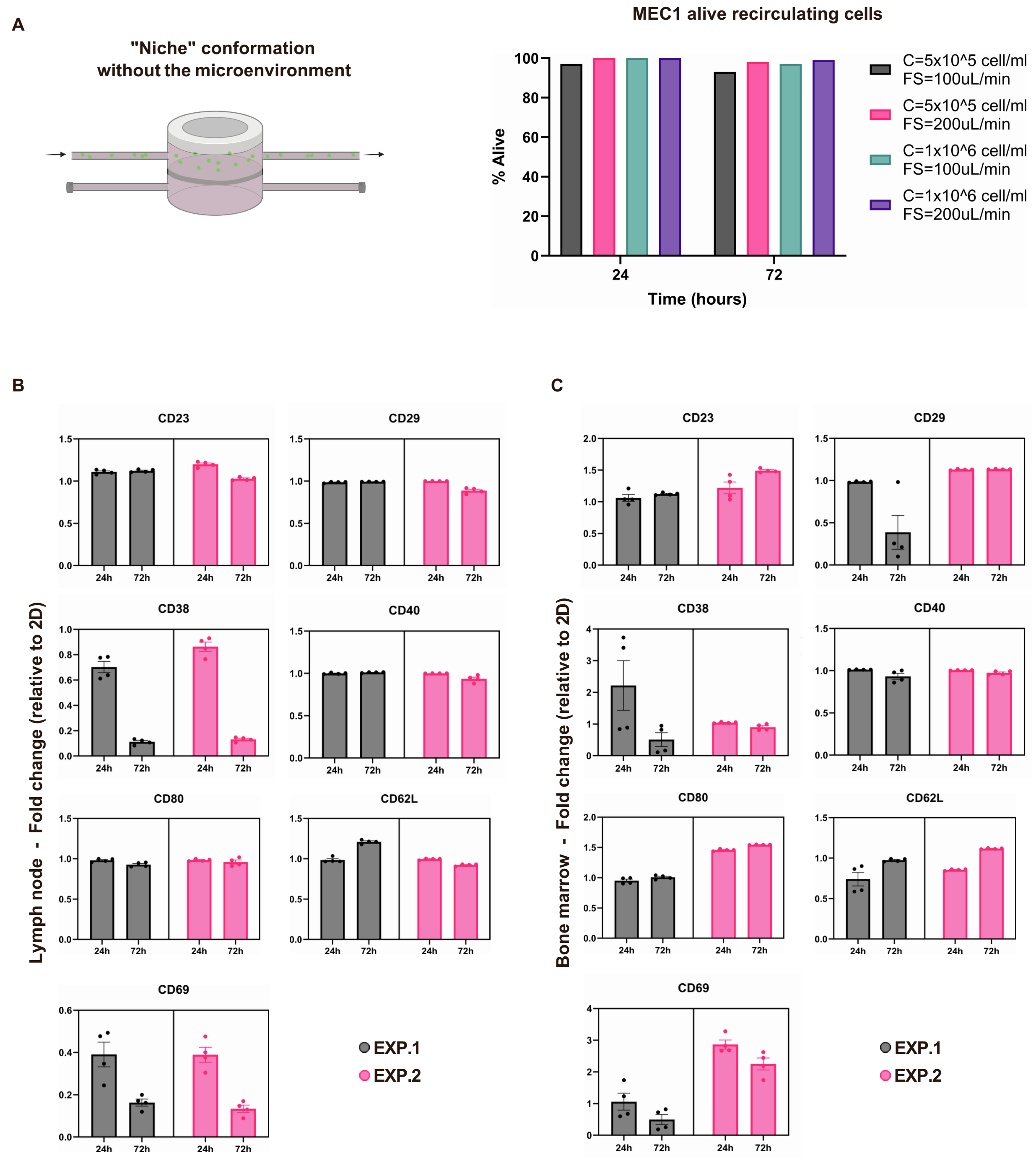

Supplementary Figure 2 - Recirculation of MEC1 cells in the system, related to Figure 7 (A) Representation of MEC1 cells recirculating in the “niche” conformation of the dynamic system without the microenvironment. The histogram shows circulating MEC1 cells viability at different cell concentration and flow rates without the microenvironment. (B) Fold change (relative to 2D condition) of different markers analyzed on MEC1 cells circulating in the dynamic sydtem with both microenvironments – bone marrow and lymph node. Data of two independent experiments (EXP.1 and EXP.2) are shown separately. For each experiment were performed 4 biological replicates. All data are represented by mean ± SEM.

A

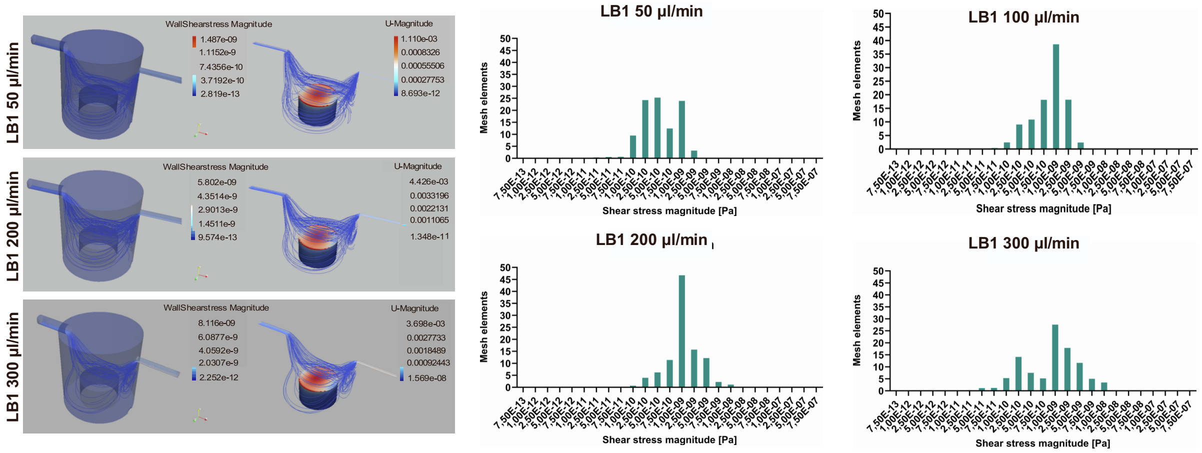

B

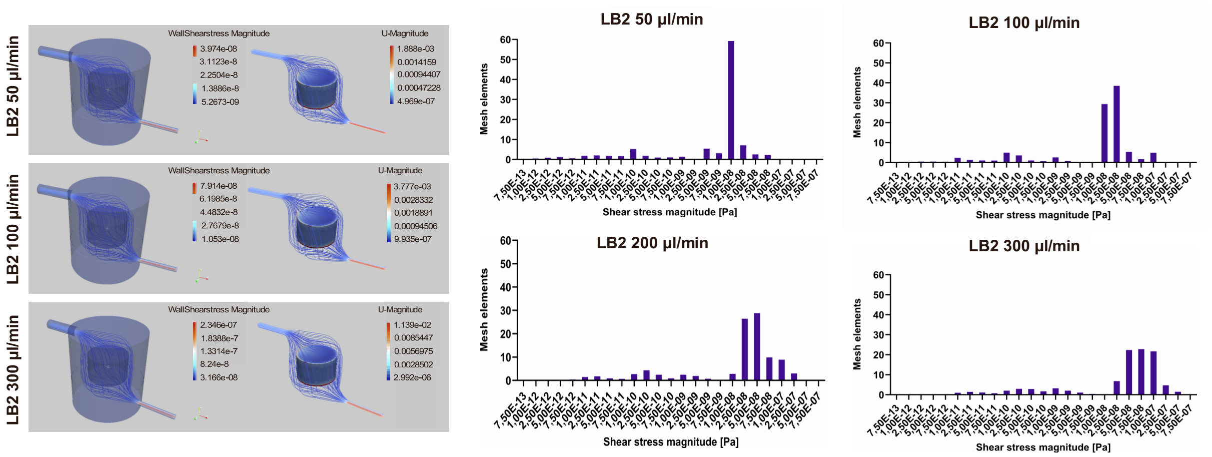

Supplementary Figure 3 - Computational Fluid Dynamic study, related to STAR methods "Computational Fluid Dynamic study (CFD)" section (A) Representation of flow lines in the bone marrow configuration (LB1) used for the bioreactors at flow rates different from the chosen ones. The colorimetric maps in both images represent the shear stress (left) and laminar flow rate (right). The histograms on the left represent the percentage of mesh elements (y-axis), normalized on the total number of the mesh elements related to the scaffold, characterized by a certain shear stress magnitude (x-axis, expressed in Pa) for LB1 at all flow rates. (B) Representation of flow lines in the lymph node configuration (LB2) used for the bioreactors at flow rates different from the chosen ones. The colorimetric maps in both images represent the shear stress (left) and laminar flow rate (right). The histograms on the left represent the percentage of mesh elements (y-axis), normalized on the total number of the mesh elements related to the scaffold, characterized by a certain shear stress magnitude (x-axis, expressed in Pa) for LB2 at all flow rates.

A

Bone Marrow	EXP.1 2D	EXP.1 24h	EXP.1 72h	EXP.2 2D	EXP.2 24h	EXP.2 72h
**IgM**	5713,45	3263,9975	3636,86	4376,88	17,7925	4247,5525
**IgD**	26016,07	24810,4625	17636,055	16525,24	0,6325	17989,6525
**CD29**	59238,16	13496,51	5689,5075	9099,6	98,315	16599,11
**CD49d**	24411,25	29323,65	26087,8	31389,34	77,72	28690,885
**CD38**	29835,25	31673,245	29760,0675	84809,27	8,7575	42017,625
**CD44**	47320,38	43186,37	27674,8175	42486,62	93,9975	29261,96
**CD40**	20958,2	37306,0325	16205,405	30221,43	99,385	18627,1175
**CD80**	25771,33	22743,025	27188,69	15749,12	89,89	34443,8575
**CD45**	26203,7	26192,0025	22351,16	41687,79	82,165	24694,0025
**CXCR4**	3734,54	5010,1125	4066,03	6193,17	20,44	4850,3825
**CCR7**	17842,02	18253,8575	17222,2375	21214,8	11,0025	19008,07
**CD69**	20851,49	20634,11	18321,7425	23592,9	25,88	18607,285
**CD23**	129048,02	150448,71	148565,915	148312,02	78,33	102356,04
**CD62L**	16293,49	9833,2975	16468,555	23894,45	61,0125	43379,3025

B

Lymph Node	EXP.1 2D	EXP.1 24h	EXP.1 72h	EXP.2 2D	EXP.2 24h	EXP.2 72h
**IgM**	9142,16	3327,41	4004,4625	6509,1	5672,92	3881,18
**IgD**	15739,23	35919,195	21038,7125	15155,32	16895,1975	18698,06
**CD29**	60886,2	12250,76	15231,005	39052,75	33210,355	8280,245
**CD49d**	38001,93	35668,1475	44880,14	38021,01	31303,005	29014,57
**CD38**	32474,07	29502,6225	29206,49	28028,86	25947,7725	26075,9375
**CD44**	30579,11	31131,35	19917,6675	213491,17	244533,315	23567,3425
**CD40**	19515,66	21571,8425	13464,3575	104447,41	143572,81	14600,1025
**CD80**	29601,01	21788,465	17087,46	29043,97	21524,4025	16095,4425
**CD45**	26242,6	20266,7225	21738,425	34207,05	38899,91	24543,02
**CXCR4**	3125,24	4679,6125	6400,85	35325,43	74773,7725	19825,1525
**CCR7**	17539,15	17366,7275	17874,43	22169	23738,12	20430,365
**CD69**	20714,79	18991,8625	18011,1325	19919,72	19073,245	19228,8725
**CD23**	167758,41	144428,0675	193795,29	125147,46	129598,66	112524,7825
**CD62L**	13620,56	11193,01	31932,67	89535,38	60702,49	34426,58

Supplementary Table 1 - Mean fluorescence intensities of flow cytometry analysis, related to Figure 7 Table reporting all the mean fluorescence intensities of the MEC1 cells recirculating in the bone marrow (A) and lymph node (B) systems. The reported values refer to the gated populations positive for the different markers displayed in Fig. 6D. Gates have been designed on FMO controls. MFI values in each experiment (EXP.1 and EXP.2) refers to the mean of the biological replicates MFI (n=4).
